# Genetically predicted causal effects of gut microbiota on spinal pain: a two-sample Mendelian randomization analysis

**DOI:** 10.3389/fmicb.2024.1357303

**Published:** 2024-03-25

**Authors:** Shuangwei Hong, Longhao Chen, Xingchen Zhou, Yuanshen Huang, Yu Tian, Huijie Hu, Bei Yu, Hongjiao Wu, Chao Yang, Zhizhen Lv, Lijiang Lv

**Affiliations:** ^1^The Third Affiliated Hospital of Zhejiang Chinese Medical University (Zhongshan Hospital of Zhejiang Province), Hangzhou, Zhejiang, China; ^2^The Third School of Clinical Medicine (School of Rehabilitation Medicine), Zhejiang Chinese Medical University, Hangzhou, Zhejiang, China; ^3^Hangzhou Hospital of Traditional Chinese Medicine, Zhejiang Chinese Medical University, Hangzhou, Zhejiang, China; ^4^Research Institute of Tuina (Spinal disease), Zhejiang Chinese Medical University, Hangzhou, Zhejiang, China

**Keywords:** spinal pain, neck pain, thoracic spine pain, low back pain, back pain, gut microbiota, Mendelian randomization, causality

## Abstract

**Background:**

Observational studies have hinted at a correlation between the gut microbiota and spinal pain (SP). However, the impact of the gut microbiota on SP remains inconclusive.

**Methods:**

In this study, we employed a two-sample Mendelian randomization (MR) analysis to explore the causal relationship between the gut microbiota and SP, encompassing neck pain (NP), thoracic spine pain (TSP), low back pain (LBP), and back pain (BP). The compiled gut microbiota data originated from a genome-wide association study (GWAS) conducted by the MiBioGen consortium (*n* = 18,340). Summary data for NP were sourced from the UK Biobank, TSP from the FinnGen Biobank, and LBP from both the UK Biobank and FinnGen Biobank. Summary data for BP were obtained from the UK Biobank. The primary analytical approach for assessing causal relationships was the Inverse Variance Weighted (IVW) method, supplemented by various sensitivity analyses to ensure result robustness.

**Results:**

The IVW analysis unveiled 37 bacterial genera with a potential causal relationship to SP. After Benjamini-Hochberg corrected test, four bacterial genera emerged with a strong causal relationship to SP. Specifically, *Oxalobacter* (OR: 1.143, 95% CI 1.061–1.232, *P* = 0.0004) and *Tyzzerella 3* (OR: 1.145, 95% CI 1.059–1.238, *P* = 0.0007) were identified as risk factors for LBP, while *Ruminococcaceae UCG011* (OR: 0.859, 95% CI 0.791–0.932, *P* = 0.0003) was marked as a protective factor for LBP, and *Olsenella* (OR: 0.893, 95% CI 0.839–0.951, *P* = 0.0004) was recognized as a protective factor for low back pain or/and sciatica. No significant heterogeneity or horizontal pleiotropy was observed through alternative testing methods.

**Conclusion:**

This study establishes a causal relationship between the gut microbiota and SP, shedding light on the “gut-spine” axis. These findings offer novel perspectives for understanding the etiology of SP and provide a theoretical foundation for potential interventions targeting the gut microbiota to prevent and treat SP.

## 1 Introduction

Spinal pain (SP) manifests as a prevalent symptom in various spine-related disorders, encompassing neck pain (NP) and back pain (BP), further categorized into thoracic spine pain (TSP) and low back pain (LBP). The prevalence in the general population is noteworthy, with NP affecting approximately 44%, TSP 15%, and LBP 56% (Manchikanti et al., 2004). The impact of SP on daily life and occupational functions surpasses that of other musculoskeletal disorders due to the spine’s role as the central axis and core support of the human skeleton (Haldeman et al., 2012). The 2015 Global Burden of Disease study underscored SP as a paramount contributor to disability globally, especially affecting individuals aged 25–64 (GBD 2015 Disease and Injury Incidence and Prevalence Collaborators, 2016). Consequently, healthcare costs for SP treatment soared, reaching an estimated $134.5 billion in the United States in 2020 (de Luca et al., 2023). The pressing need for effective prevention and treatment strategies for SP is a critical concern in the realm of global public health.

The gut microbiota, comprising over 10 trillion microorganisms, exists harmoniously within the human intestinal tract, outnumbering human cells tenfold (Clark and Coopersmith, 2007; Zhang et al., 2015). The colon, serving as the primary habitat, hosts an astounding 10^∧^11–10^∧^12 microorganisms per milliliter (Ley et al., 2006). This rich, diverse, and stable microbiota plays a crucial role in sustaining various physiological functions, including nutrient metabolism, xenobiotic and drug metabolism, immunomodulation, and antimicrobial defense (Jandhyala et al., 2015). Mounting evidence links ecological destabilization of the gut microbiota to the development of various diseases, including intestinal (Quaglio et al., 2022), hepatic (Chassaing et al., 2014), metabolic (Lin et al., 2020), allergic (Akagawa and Kaneko, 2022), and autoimmune diseases (Yang et al., 2023).

Recent studies propose a potential relationship between the gut microbiota and the development of SP. Patients with NP exhibited higher levels of *Bacteroidetes* and *Proteobacteria* and significantly lower levels of *Firmicutes* compared to healthy controls (Qin, 2022). Similarly, individuals with BP showed a distinct gut microbiota composition, characterized by elevated concentrations of *Adlercreutzia*, *Roseburia*, and *Uncl. Christensenellaceae* (Dekker Nitert et al., 2020). These findings underscore a compelling relationship between gut microbiota and SP. While degenerative spinal diseases traditionally bear the blame for SP, a notable shift toward a younger demographic experiencing SP suggests alternative causative factors beyond structural spinal degeneration (Lei et al., 2015). Consequently, gut microbiota dysbiosis emerges as a significant potential contributor to SP.

In light of these observations, MR offers a promising avenue for exploring causal relationships. MR, first proposed by Smith and Ebrahim (2003), utilizes genetic variation as an instrumental variable, enabling the elimination of confounding bias and reverse causation (Emdin et al., 2017; Soremekun et al., 2022). In this study, we collected data on gut microbiota and SP from the genome-wide association study (GWAS) database, employing MR analysis to discern the causal relationship between them. The study aims to provide innovative insights for the prevention and treatment of SP by unraveling the intricate interplay between gut microbiota dynamics and SP.

## 2 Materials and methods

### 2.1 Study design

**FIGURE 1:**
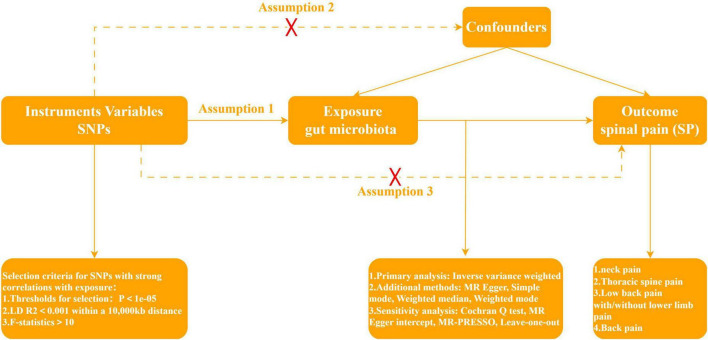
Three assumptions and flowchart for Mendelian randomization study.

Mendelian Randomization, a pivotal method for evaluating causal relationships between exposure and outcome, stands as the cornerstone of our investigative framework. In quest of discerning whether gut microbiota assumes a protective or facilitative role in SP development, our study strategically designates gut microbiota as the exposure variable, with four SP types—NP, TSP, LBP, and BP—comprising the outcome variables. According to Davies et al. (2018), the implementation of a two-sample MR protocol necessitated strict adherence to three fundamental assumptions. First, the instrumental variables, genetic variation, should exhibit a robust relationship with gut microbiota. Second, genetic variation must remain unlinked to confounding factors, excluding those pertaining to gut microbiota and the four SP types. Third, the effects of genetic variation on the four SP types must be channeled through a singular pathway—namely, gut microbiota—with the exclusion of any alternative pathways. In Figure 1, the specific flowchart detailing the MR analysis is presented.

### 2.2 Exposure data

The summary statistics for gut microbiota utilized in this investigation originated from a GWAS conducted by the MiBioGen consortium in 2021 (Kurilshikov et al., 2021). The study encompassed a substantial cohort of 18,340 individuals, with over 70% representing European ancestry (*n* = 13,266). Within this cohort, participants were stratified into 24 distinct groups based on factors such as background, sex, and age. The composition of their fecal microbiota was meticulously examined through the application of 16S rRNA gene sequencing. This comprehensive analysis led to the identification of 211 taxa, spanning across 131 genera, 35 families, 20 orders, 16 classes, and 9 phyla. Recognizing the significance of genus-level precision in characterizing the microbiome, we opted to exclusively retain data at the genus level for subsequent phases of our study and detailed analysis. This strategic decision ensures a more granular and accurate exploration of each potentially pathogenic microbiome.

### 2.3 Outcome data

All GWAS summary statistics pertaining to SP were meticulously sourced from the EU Open GWAS platform.^1^ This comprehensive repository comprises data packages for the four primary types of SP: NP, TSP, LBP, and BP. After a thorough review of pertinent datasets, eight were judiciously selected for our analysis. Specifically, two GWAS summary statistics for NP, considering disease durations of 1 month and 3+ months, were incorporated into our study, both sourced from the UK Biobank (ukb-e). The GWAS summary statistics for TSP were obtained from the FinnGen Biobank. Similarly, the GWAS summary statistics for LBP were procured from the FinnGen Biobank. Recognizing that patients with LBP often exhibit radiating pain in the lower limbs (Koes et al., 2006), we expanded our dataset to include two GWAS summary statistics: one for low back pain with radicular pain from the UK Biobank (ukb-e) and another for low back pain with sciatica from the FinnGen Biobank. For the BP category, two GWAS summary statistics for BP, comparing disease durations of 1 month versus 3+ months, were selected from the UK Biobank (ukb-e). For a detailed breakdown of outcome summary statistics, please refer to Supplementary Table 1.

### 2.4 Instrumental variable (IV)

In order to ensure the precision and robustness of the study outcomes, we incorporated single nucleotide polymorphisms (SNPs) demonstrating robust relationships with the exposure factors. While the conventional threshold for controlling the false positive rate is typically set at *P* < 5e−08, this study opted for a more inclusive threshold of *P* < 1e−05 (Xie et al., 2023). This adjustment was made to augment the pool of screened SNPs, thereby enhancing result credibility, given the limited number of SNPs obtained under the more stringent condition. Subsequently, to acquire mutually independent instrumental variables, SNPs within a 10,000 kb clumping window were excluded to mitigate Linkage Disequilibrium (LD), specifically retaining SNPs with an R^2^ < 0.001. Concurrently, F-statistics of the SNPs were computed, leading to the exclusion of weak instrumental variables characterized by *F* < 10 (Burgess and Thompson, 2011). Finally, a meticulous harmonization process was applied to align the effector alleles of the exposure and outcome SNPs. This involved the exclusion of alleles that were incompatible (e.g., A/C paired with A/G) or exhibited intermediate allele frequencies.

### 2.5 Statistical analysis

The causal relationship between the gut microbiota and SP was primarily analyzed using the Inverse Variance Weighted (IVW) method. A potential causal relationship between the gut microbiota and SP was considered when the IVW result yielded a *P* < 0.05. Following multiple testing correction of the IVW results using the Benjamini-Hochberg False Discovery Rate (FDR) method, gut microbiota with *P* < 0.05 were deemed to exhibit a significant causal relationship with SP. However, the validity of this conclusion relies on the absence of horizontal pleiotropy and heterogeneity (Burgess et al., 2016). Therefore, various additional methods were incorporated to scrutinize the potential presence or absence of horizontal pleiotropy and heterogeneity.

Initially, Cochrane’s Q test was employed to assess heterogeneity in the IVs. A significance level of *P* < 0.05 denoted heterogeneity, necessitating the use of the random-effects IVW model; conversely, the fixed-effects IVW model was applied (Greco et al., 2015). In the IVW regression, where the intercept term is not considered, results become inaccurate in the presence of horizontal pleiotropy (Burgess et al., 2017). To address this, the MR-Egger regression method and the Weighted Median (WM) method were employed to correct for horizontal pleiotropy. MR-Egger assumes that the IVs of horizontal pleiotropy is greater than 50%, while WM assumes it to be less than 50%, both re-estimating results accordingly (Bowden et al., 2016). Both methods complemented the IVW method, correcting for bias introduced by horizontal pleiotropy and enhancing result credibility (Bowden et al., 2015; Slob and Burgess, 2020). MR-Egger and MR Pleiotropy RESidual Sum and Outlier (MR-PRESSO) were employed to test horizontal pleiotropy. An MR-Egger *P*-value of less than 0.05 indicated a non-zero intercept, signifying the presence of horizontal pleiotropy. In addition to testing horizontal pleiotropy, MR-PRESSO identified and corrected for outliers, thus improving the accuracy of the analysis results. Finally, a leave-one-out sensitivity analysis was conducted to assess the stability of the MR results and detect the presence of outliers in the SNPs.

All the aforementioned statistical analyses were performed using the R program (version 4.3.1), with the “VariantAnnotation,” “mrcieu/gwasglue,” “mrcieu/ieugwasr,” and “MRCIEU/TwoSampleMR” packages loaded and executed. The specific MR analysis codes are detailed in the Supplementary material.

## 3 Results

### 3.1 SNP selection

From a pool of 119 bacterial genera, we meticulously screened and identified 1531 SNPs to serve as IVs in our analysis. Crucially, all of these SNPs exhibited F-statistics surpassing 10, with values ranging from 14.58 to 88.42. This range signifies a robust F-statistic profile, indicating the absence of bias arising from weak IVs. This stringent selection process ensures the reliability and strength of the IVs utilized in our study.

### 3.2 Causal influence of gut microbiota on the risk of spinal pain

#### 3.2.1 Causal influence of gut microbiota on the risk of neck pain

**FIGURE 2:**
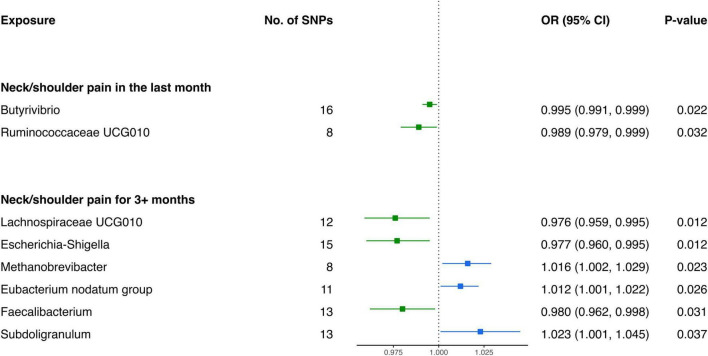
Forest plot of causality between gut microbiota and risk of neck pain.

Based on the outcomes of the IVW analysis, we identified two bacterial genera demonstrating protective effects against NP in the last month (Figure 2 and Table 1): *Ruminococcaceae UCG010* (OR: 0.989, 95% CI 0.979–0.999, *P* = 0.032) and *Butyrivibrio* (OR: 0.995, 95% CI 0.991–0.999, *P* = 0.022).

**TABLE 1:** MR results of causal effects between gut microbiome and neck pain.

Outcome	Exposure	nSNP	MR method	SE	OR (95% CI)	*p*-value
Neck/shoulder pain in the last month	Butyrivibrio	16	MR Egger	0.009	0.996 (0.978, 1.014)	0.673
			Weighted median	0.003	0.997 (0.991, 1.002)	0.203
			IVW	0.002	0.995 (0.991, 0.999)	0.022
			Simple mode	0.004	0.997 (0.988, 1.005)	0.458
			Weighted mode	0.004	0.997 (0.989, 1.005)	0.477
	Ruminococcaceae UCG010	8	MR Egger	0.019	0.981 (0.946, 1.019)	0.363
			Weighted median	0.007	0.986 (0.974, 0.999)	0.039
			IVW	0.005	0.989 (0.979, 0.999)	0.032
			Simple mode	0.009	0.987 (0.969, 1.004)	0.181
			Weighted mode	0.009	0.986 (0.968, 1.005)	0.186
Neck/shoulder pain for 3+ months	Lachnospiraceae UCG010	12	MR Egger	0.026	0.952 (0.904, 1.001)	0.085
			Weighted median	0.012	0.980 (0.957, 1.004)	0.104
			IVW	0.009	0.976 (0.959, 0.995)	0.012
			Simple mode	0.021	0.986 (0.946, 1.029)	0.533
			Weighted mode	0.017	0.984 (0.951, 1.018)	0.358
	Escherichia-Shigella	15	MR Egger	0.026	1.004 (0.953, 1.058)	0.878
			Weighted median	0.012	0.976 (0.953, 1.001)	0.056
			IVW	0.009	0.977 (0.960, 0.995)	0.012
			Simple mode	0.024	0.959 (0.914, 1.006)	0.110
			Weighted mode	0.022	1.009 (0.966, 1.053)	0.708
	Methanobrevibacter	8	MR Egger	0.026	1.041 (0.990, 1.094)	0.170
			Weighted median	0.008	1.014 (0.998, 1.032)	0.091
			IVW	0.007	1.016 (1.002, 1.029)	0.023
			Simple mode	0.014	1.016 (0.988, 1.045)	0.305
			Weighted mode	0.014	1.014 (0.987, 1.042)	0.338
	Eubacterium nodatum group	11	MR Egger	0.023	1.027 (0.981, 1.074)	0.284
			Weighted median	0.007	1.013 (0.999, 1.027)	0.065
			IVW	0.005	1.012 (1.001, 1.022)	0.026
			Simple mode	0.012	1.017 (0.992, 1.041)	0.212
			Weighted mode	0.012	1.016 (0.992, 1.039)	0.220
	Faecalibacterium	13	MR Egger	0.019	1.017 (0.979, 1.056)	0.415
			Weighted median	0.014	0.985 (0.959, 1.012)	0.265
			IVW	0.009	0.980 (0.962, 0.998)	0.031
			Simple mode	0.021	0.985 (0.945, 1.026)	0.480
			Weighted mode	0.020	0.983 (0.946, 1.023)	0.416
	Subdoligranulum	13	MR Egger	0.029	1.027 (0.969, 1.088)	0.394
			Weighted median	0.015	1.018 (0.989, 1.048)	0.229
			IVW	0.011	1.023 (1.001, 1.045)	0.037
			Simple mode	0.023	1.017 (0.973, 1.064)	0.467
			Weighted mode	0.021	1.017 (0.976, 1.060)	0.441

MR, Mendelian randomisation; SE, standard error; IVW, inverse variance weighted; OR, odds ratio; CI, confidence interval.

Furthermore, the IVW estimates indicated that *Methanobrevibacter* (OR: 1.016, 95% CI 1.002–1.029, *P* = 0.023), *Eubacterium nodatum group* (OR: 1.012, 95% CI 1.001–1.022, *P* = 0.026), and *Subdoligranulum* (OR: 1.023, 95% CI 1.001–1.045, *P* = 0.037) were positively associated with the risk of NP for 3+ months (Figure 2). In contrast, the genetically predicted abundance of *Escherichia-Shigella* (OR: 0.977, 95% CI 0.960–0.995, *P* = 0.012), *Faecalibacterium* (OR: 0.980, 95% CI 0.962–0.998, *P* = 0.031), and *Lachnospiraceae UCG010* (OR: 0.976, 95% CI 0.959–0.995, *P* = 0.012) exhibited a negative association with the risk of NP for 3+ months (Table 1).

#### 3.2.2 Causal influence of gut microbiota on the risk of thoracic spine pain

**FIGURE 3:**
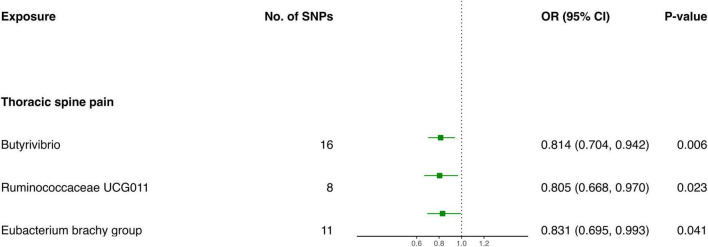
Forest plot of causality between gut microbiota and risk of thoracic spine pain.

The IVW screening revealed three bacterial genera exhibiting a causal relationship with TSP (Figure 3 and Table 2). The outcomes indicated a genetically predicted low relative abundance for *Butyrivibrio* (OR: 0.814, 95% CI 0.704–0.942, *P* = 0.006), *Ruminococcaceae UCG011* (OR: 0.805, 95% CI 0.668–0.970, *P* = 0.023), and *Eubacterium brachy group* (OR: 0.831, 95% CI 0.695–0.993, *P* = 0.041), which were associated with a reduced risk of TSP.

**TABLE 2:** MR results of causal effects between gut microbiome and thoracic spine pain.

Outcome	Exposure	nSNP	MR method	SE	OR (95% CI)	*p*-value
Thoracic spine pain	Butyrivibrio	16	MR Egger	0.299	1.333 (0.741, 2.398)	0.352928
			Weighted median	0.100	0.831 (0.682, 1.011)	0.064856
			IVW	0.074	0.814 (0.704, 0.942)	0.005597
			Simple mode	0.165	0.796 (0.576, 1.100)	0.186943
			Weighted mode	0.148	0.816 (0.610, 1.090)	0.189155
	Ruminococcaceae UCG011	8	MR Egger	0.476	1.254 (0.493, 3.190)	0.652
			Weighted median	0.125	0.816 (0.639, 1.042)	0.103
			IVW	0.095	0.805 (0.668, 0.970)	0.023
			Simple mode	0.193	0.767 (0.526, 1.120)	0.212
			Weighted mode	0.184	0.813 (0.566, 1.167)	0.298
	Eubacterium brachy group	11	MR Egger	0.329	0.627 (0.329, 1.195)	0.190
			Weighted median	0.127	0.839 (0.653, 1.076)	0.167
			IVW	0.091	0.831 (0.695, 0.993)	0.041
			Simple mode	0.198	0.781 (0.530, 1.151)	0.240
			Weighted mode	0.218	0.778 (0.508, 1.192)	0.276

MR, mendelian randomisation; SE, standard error; IVW, inverse variance weighted; OR, odds ratio; CI, confidence interval.

#### 3.2.3 Causal influence of gut microbiota on the risk of low back pain

**FIGURE 4:**
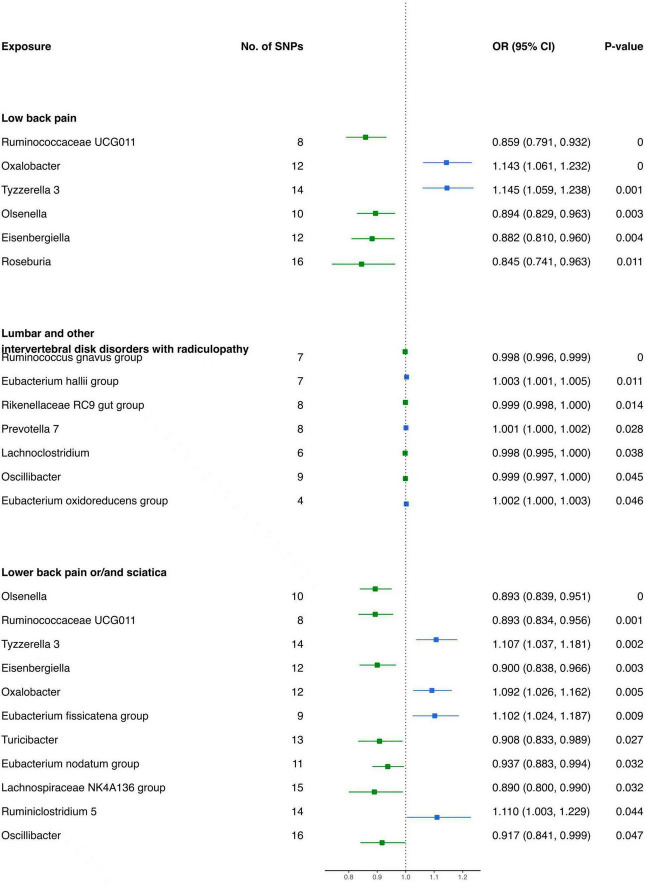
Forest plot of causality between gut microbiota and risk of low back pain with/without lower limb pain.

The results of the IVW analysis delineate distinct associations between bacterial genera and the risk of LBP. Genetically predicted *Oxalobacter* (OR: 1.143, 95% CI 1.061–1.232, *P* < 0.001) and *Tyzzerella 3* (OR: 1.145, 95% CI 1.059–1.238, *P* < 0.001) were found to be correlated with an elevated risk of LBP (Figure 4). In contrast, *Ruminococcaceae UCG011* (OR: 0.859, 95% CI 0.791–0.932, *P* < 0.001), *Olsenella* (OR: 0.894, 95% CI 0.829–0.963, *P* = 0.003), *Eisenbergiella* (OR: 0.882, 95% CI 0.810–0.960, *P* = 0.004), and *Roseburia* (OR: 0.845, 95% CI 0.741–0.963, *P* = 0.011) were associated with a reduced risk of LBP (Table 3).

**TABLE 3:** MR results of causal effects between gut microbiome and low back pain with/without lower limb pain.

Outcome	Exposure	nSNP	MR method	SE	OR (95% CI)	*p*-value
Low back pain	Ruminococcaceae UCG011	8	MR Egger	0.209	1.071 (0.710, 1.614)	0.756
			Weighted median	0.056	0.841 (0.753, 0.939)	0.002
			IVW	0.042	0.859 (0.791, 0.932)	0.000
			Simple mode	0.095	0.818 (0.679, 0.986)	0.073
			Weighted mode	0.098	0.818 (0.675, 0.991)	0.079
	Oxalobacter	12	MR Egger	0.167	1.109 (0.799, 1.540)	0.550
			Weighted median	0.051	1.117 (1.011, 1.235)	0.030
			IVW	0.038	1.143 (1.061, 1.232)	0.000
			Simple mode	0.086	1.051 (0.889, 1.243)	0.571
			Weighted mode	0.084	1.055 (0.895, 1.244)	0.534
	Tyzzerella 3	14	MR Egger	0.233	1.172 (0.742, 1.850)	0.509
			Weighted median	0.053	1.134 (1.022, 1.257)	0.017
			IVW	0.040	1.145 (1.059, 1.238)	0.001
			Simple mode	0.093	1.127 (0.938, 1.353)	0.224
			Weighted mode	0.084	1.130 (0.958, 1.332)	0.170
	Olsenella	10	MR Egger	0.122	1.003 (0.789, 1.274)	0.983
			Weighted median	0.053	0.909 (0.820, 1.008)	0.071
			IVW	0.038	0.894 (0.829, 0.963)	0.003
			Simple mode	0.079	0.942 (0.807, 1.101)	0.475
			Weighted mode	0.070	0.912 (0.796, 1.045)	0.218
	Eisenbergiella	12	MR Egger	0.320	1.136 (0.607, 2.126)	0.697
			Weighted median	0.058	0.897 (0.800, 1.005)	0.061
			IVW	0.043	0.882 (0.810, 0.960)	0.004
			Simple mode	0.089	0.901 (0.757, 1.073)	0.268
			Weighted mode	0.084	0.910 (0.772, 1.073)	0.288
	Roseburia	16	MR Egger	0.193	0.927 (0.635, 1.354)	0.701
			Weighted median	0.095	0.826 (0.686, 0.996)	0.045
			IVW	0.067	0.845 (0.741, 0.963)	0.011
			Simple mode	0.169	0.742 (0.532, 1.034)	0.099
			Weighted mode	0.174	0.754 (0.536, 1.061)	0.126
Lumbar and other intervertebral disk disorders with radiculopathy	Ruminococcus gnavus group	7	MR Egger	0.005	0.998 (0.988, 1.007)	0.671
			Weighted median	0.001	0.997 (0.996, 0.999)	0.001
			IVW	0.001	0.998 (0.996, 0.999)	0.000
			Simple mode	0.001	0.997 (0.995, 0.999)	0.050
			Weighted mode	0.001	0.997 (0.995, 0.999)	0.044
	Eubacterium hallii group	7	MR Egger	0.019	0.993 (0.957, 1.030)	0.714
			Weighted median	0.001	1.002 (1.000, 1.005)	0.097
			IVW	0.001	1.003 (1.001, 1.005)	0.011
			Simple mode	0.002	1.003 (0.999,1.007)	0.197
			Weighted mode	0.002	1.003 (0.999, 1.007)	0.218
	Rikenellaceae RC9 gut group	8	MR Egger	0.004	0.997 (0.990, 1.004)	0.384
			Weighted median	0.001	0.999 (0.997, 1.000)	0.011
			IVW	0.000	0.999 (0.998, 1.000)	0.014
			Simple mode	0.001	0.998 (0.997, 1.000)	0.118
			Weighted mode	0.001	0.998 (0.997, 1.000)	0.126
	Prevotella 7	8	MR Egger	0.005	1.001 (0.992, 1.010)	0.808
			Weighted median	0.001	1.001 (1.000, 1.002)	0.097
			IVW	0.000	1.001 (1.000, 1.002)	0.028
			Simple mode	0.001	1.001 (0.999, 1.003)	0.304
			Weighted mode	0.001	1.001 (0.999, 1.003)	0.291
	Lachnoclostridium	6	MR Egger	0.013	1.003 (0.979, 1.028)	0.804
			Weighted median	0.001	0.998 (0.995, 1.001)	0.116
			IVW	0.001	0.998 (0.995, 1.000)	0.038
			Simple mode	0.002	0.999 (0.995, 1.003)	0.670
			Weighted mode	0.002	0.999 (0.995, 1.003)	0.677
	Oscillibacter	9	MR Egger	0.010	0.992 (0.972, 1.013)	0.482
			Weighted median	0.001	0.999 (0.997, 1.000)	0.093
			IVW	0.001	0.999 (0.997, 1.000)	0.045
			Simple mode	0.001	0.999 (0.996, 1.001)	0.371
			Weighted mode	0.001	0.999 (0.996, 1.001)	0.342
	Eubacterium oxidoreducens group	4	MR Egger	0.048	0.982 (0.894, 1.078)	0.736
			Weighted median	0.001	1.002 (1.000, 1.004)	0.071
			IVW	0.001	1.002 (1.000, 1.003)	0.046
			Simple mode	0.001	1.002 (1.000, 1.004)	0.198
			Weighted mode	0.001	1.002 (0.999, 1.004)	0.232
Lower back pain or/and sciatica	Olsenella	10	MR Egger	0.102	1.035 (0.848, 1.263)	0.745
			Weighted median	0.044	0.915 (0.838, 0.997)	0.044
			IVW	0.032	0.893 (0.839, 0.951)	0.000
			Simple mode	0.062	0.928 (0.822, 1.049)	0.263
			Weighted mode	0.054	0.927 (0.834, 1.030)	0.192
	Ruminococcaceae UCG011	8	MR Egger	0.174	0.846 (0.602, 1.190)	0.374
			Weighted median	0.046	0.903 (0.825, 0.988)	0.025
			IVW	0.035	0.893 (0.834, 0.956)	0.001
			Simple mode	0.063	0.897 (0.792, 1.015)	0.130
			Weighted mode	0.058	0.899 (0.802, 1.007)	0.107
	Tyzzerella 3	14	MR Egger	0.194	1.143 (0.782, 1.671)	0.504
			Weighted median	0.044	1.141 (1.046, 1.244)	0.003
			IVW	0.033	1.107 (1.037, 1.181)	0.002
			Simple mode	0.070	1.155 (1.008, 1.324)	0.059
			Weighted mode	0.070	1.155 (1.007, 1.326)	0.061
	Eisenbergiella	12	MR Egger	0.266	1.024 (0.608, 1.726)	0.930
			Weighted median	0.049	0.907 (0.823, 0.999)	0.047
			IVW	0.036	0.900 (0.838, 0.966)	0.003
			Simple mode	0.080	0.904 (0.772, 1.058)	0.234
			Weighted mode	0.082	0.905 (0.771, 1.063)	0.248
	Oxalobacter	12	MR Egger	0.140	1.098 (0.835, 1.445)	0.518
			Weighted median	0.044	1.041 (0.954, 1.136)	0.369
			IVW	0.032	1.092 (1.026, 1.162)	0.005
			Simple mode	0.068	1.015 (0.889, 1.160)	0.826
			Weighted mode	0.067	1.014 (0.889, 1.157)	0.837
	Eubacterium fissicatena group	9	MR Egger	0.199	1.297 (0.877, 1.918)	0.233
			Weighted median	0.051	1.058 (0.957, 1.170)	0.267
			IVW	0.038	1.102 (1.024, 1.187)	0.009
			Simple mode	0.075	1.068 (0.923, 1.236)	0.402
			Weighted mode	0.070	1.060 (0.924, 1.215)	0.431
	Turicibacter	13	MR Egger	0.186	0.784 (0.545, 1.128)	0.216
			Weighted median	0.058	0.904 (0.807, 1.014)	0.086
			IVW	0.044	0.908 (0.833, 0.989)	0.027
			Simple mode	0.098	0.880 (0.727, 1.066)	0.216
			Weighted mode	0.095	0.874 (0.725, 1.053)	0.182
	Eubacterium nodatum group	11	MR Egger	0.135	0.993 (0.763, 1.293)	0.960
			Weighted median	0.041	0.928 (0.856, 1.007)	0.072
			IVW	0.030	0.937 (0.883, 0.994)	0.032
			Simple mode	0.064	0.923 (0.815, 1.046)	0.237
			Weighted mode	0.067	0.925 (0.811, 1.054)	0.268
	Lachnospiraceae NK4A136 group	15	MR Egger	0.108	0.805 (0.651, 0.995)	0.067
			Weighted median	0.071	0.864 (0.752, 0.994)	0.042
			IVW	0.055	0.890 (0.800, 0.990)	0.032
			Simple mode	0.139	0.823 (0.627, 1.080)	0.181
			Weighted mode	0.082	0.865 (0.736, 1.016)	0.099
	Ruminiclostridium 5	14	MR Egger	0.129	1.060 (0.823, 1.366)	0.658
			Weighted median	0.073	1.070 (0.928, 1.234)	0.353
			IVW	0.052	1.110 (1.003, 1.229)	0.044
			Simple mode	0.104	1.093 (0.891, 1.341)	0.409
			Weighted mode	0.086	1.073 (0.907, 1.270)	0.423
	Oscillibacter	16	MR Egger	0.157	0.963 (0.709, 1.309)	0.814
			Weighted median	0.055	0.931 (0.835, 1.038)	0.199
			IVW	0.044	0.917 (0.841, 0.999)	0.047
			Simple mode	0.095	0.920 (0.764, 1.107)	0.391
			Weighted mode	0.082	0.926 (0.788, 1.087)	0.361

MR, mendelian randomisation; SE, standard error; IVW, inverse variance weighted; OR, odds ratio; CI, confidence interval.

For lumbar and other intervertebral disk disorders with radiculopathy, specific bacterial genera demonstrated distinct associations. *Prevotella 7* (OR: 1.001, 95% CI 1.000–1.002, *P* = 0.028), *Eubacterium hallii group* (OR: 1.003, 95% CI 1.001–1.005, *P* = 0.011), and *Eubacterium oxidoreducens group* (OR: 1.002, 95% CI 1.000–1.003, *P* = 0.046) were positively associated with the risk (Figure 4). Conversely, *Lachnoclostridium* (OR: 0.998, 95% CI 0.995–1.000, *P* = 0.038), *Ruminococcus gnavus group* (OR: 0.998, 95% CI 0.996–0.999, *P* < 0.001), *Rikenellaceae RC9 gut group* (OR: 0.999, 95% CI 0.998–1.000, *P* = 0.014), and *Oscillibacter* (OR: 0.999, 95% CI 0.997–1.000, *P* = 0.045) were negatively associated with the risk of these disorders (Table 3).

Moreover, for low back pain or/and sciatica, *Tyzzerella 3* (OR: 1.107, 95% CI 1.037–1.181, *P* = 0.002), *Oxalobacter* (OR: 1.092, 95% CI 1.026–1.162, *P* = 0.005), *Eubacterium fissicatena group* (OR. 1.102, 95% CI 1.024–1.187, *P* = 0.009), and *Ruminiclostridium 5* (OR: 1.110, 95% CI 1.003–1.229, *P* = 0.044) were identified as having contributory effects (Figure 4). Conversely, *Olsenella* (OR: 0.893, 95% CI 0.839–0.951, *P* < 0.001), *Ruminococcaceae UCG011* (OR: 0.893, 95% CI 0.834–0.956, *P* = 0.001), *Eisenbergiella* (OR: 0.900, 95% CI 0.838–0.966, *P* = 0.003), *Turicibacter* (OR: 0.908, 95% CI 0.833–0.989, *P* = 0.027), *Eubacterium nodatum group* (OR: 0.937, 95% CI 0.883–0.994, *P* = 0.032), *Lachnospiraceae NK4A136 group* (OR: 0.890, 95% CI 0.800–0.990, *P* = 0.032), and *Oscillibacter* (OR: 0.917, 95% CI 0.841–0.999, *P* = 0.047) were found to be protective (Table 3).

#### 3.2.4 Causal influence of gut microbiota on the risk of back pain

**FIGURE 5:**
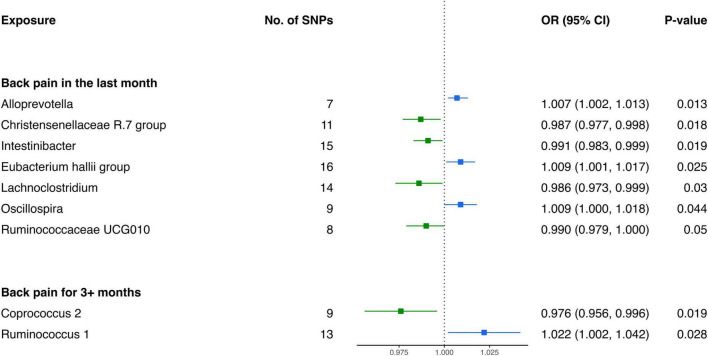
Forest plot of causality between gut microbiota and risk of back pain.

Analysis from the IVW test has revealed distinctive associations between specific bacterial genera and the risk of BP across varying durations. For BP in the last month, genetically predicted relative abundance of *Alloprevotella* (OR: 1.007, 95% CI 1.002–1.013, *P* = 0.013), *Oscillospira* (OR: 1.009, 95% CI 1.000–1.018, *P* = 0.044), and *Eubacterium hallii group* (OR: 1.009, 95% CI 1.001–1.017, *P* = 0.025) exhibited positive relationships with increased risk (Figure 5). Contrarily, *Christensenellaceae R.7 group* (OR: 0.987, 95% CI 0.977–0.998, P = 0.018), *Intestinibacter* (OR: 0.991, 95% CI 0.983–0.999, *P* = 0.019), *Ruminococcaceae UCG010* (OR: 0.990, 95% CI 0.979–1.000, *P* = 0.050), and *Lachnoclostridium* (OR: 0.986, 95% CI 0.973–0.999, *P* = 0.030) were inversely associated with the risk of BP in the last month (Table 4).

**TABLE 4:** MR results of causal effects between gut microbiome and back pain.

Outcome	Exposure	nSNP	MR method	SE	OR (95% CI)	*p*-value
Back pain in the last month	Alloprevotella	7	MR Egger	0.028	1.034 (0.979, 1.092)	0.284
			Weighted median	0.004	1.006 (0.999, 1.014)	0.114
			IVW	0.003	1.007 (1.002, 1.013)	0.013
			Simple mode	0.006	1.007 (0.996, 1.018)	0.255
			Weighted mode	0.006	1.006 (0.995, 1.017)	0.313
	Christensenellaceae R.7 group	11	MR Egger	0.017	0.982 (0.951, 1.015)	0.312
			Weighted median	0.007	0.987 (0.973, 1.001)	0.079
			IVW	0.005	0.987 (0.977, 0.998)	0.018
			Simple mode	0.011	0.988 (0.967, 1.011)	0.325
			Weighted mode	0.011	0.988 (0.968, 1.009)	0.291
	Intestinibacter	15	MR Egger	0.013	0.966 (0.942, 0.991)	0.018
			Weighted median	0.005	0.989 (0.978, 0.999)	0.039
			IVW	0.004	0.991 (0.983, 0.999)	0.019
			Simple mode	0.011	0.987 (0.967, 1.008)	0.249
			Weighted mode	0.009	0.987 (0.970, 1.005)	0.187
	Eubacterium hallii group	16	MR Egger	0.008	1.007 (0.991, 1.023)	0.435
			Weighted median	0.006	1.009 (0.999, 1.020)	0.087
			IVW	0.004	1.009 (1.001, 1.017)	0.025
			Simple mode	0.010	1.006 (0.986, 1.026)	0.573
			Weighted mode	0.009	1.009 (0.992, 1.026)	0.336
	Lachnoclostridium	14	MR Egger	0.023	1.021 (0.977, 1.067)	0.378
			Weighted median	0.008	0.980 (0.965, 0.995)	0.010
			IVW	0.007	0.986 (0.973, 0.999)	0.030
			Simple mode	0.013	0.973 (0.949, 0.998)	0.056
			Weighted mode	0.013	0.974 (0.949, 1.000)	0.074
	Oscillospira	9	MR Egger	0.019	0.968 (0.933, 1.005)	0.137
			Weighted median	0.006	1.009 (0.997, 1.021)	0.155
			IVW	0.005	1.009 (1.000, 1.018)	0.044
			Simple mode	0.011	1.014 (0.992, 1.036)	0.262
			Weighted mode	0.010	1.000 (0.980, 1.021)	0.993
	Ruminococcaceae UCG010	8	MR Egger	0.020	0.988 (0.950, 1.027)	0.560
			Weighted median	0.007	0.990 (0.977, 1.003)	0.125
			IVW	0.005	0.990 (0.979, 1.000)	0.050
			Simple mode	0.010	0.990 (0.970, 1.010)	0.348
			Weighted mode	0.010	0.990 (0.970, 1.010)	0.352
Back pain for 3+ months	Coprococcus 2	9	MR Egger	0.057	0.892 (0.798, 0.998)	0.086
			Weighted median	0.014	0.971 (0.944, 0.999)	0.041
			IVW	0.010	0.976 (0.956, 0.996)	0.019
			Simple mode	0.021	0.962 (0.924, 1.002)	0.103
			Weighted mode	0.022	0.962 (0.922, 1.004)	0.117
	Ruminococcus 1	13	MR Egger	0.027	1.016 (0.963, 1.071)	0.577
			Weighted median	0.013	1.018 (0.992, 1.045)	0.167
			IVW	0.010	1.022 (1.002, 1.042)	0.028
			Simple mode	0.022	1.009 (0.966, 1.053)	0.698
			Weighted mode	0.020	1.025 (0.984, 1.067)	0.256

MR, mendelian randomisation; SE, standard error; IVW, inverse variance weighted; OR, odds ratio; CI, confidence interval.

Furthermore, in the context of BP for 3+ months, two bacterial genera exhibited causal relationships. The genetically predicted relative abundance of *Ruminococcus 1* (OR: 1.022, 95% CI 1.002–1.042, *P* = 0.028) was linked to an increased risk of BP for 3+ months. Conversely, *Coprococcus 2* (OR: 0.976, 95% CI 0.956–0.996, *P* = 0.019) demonstrated a negative association, suggesting a potential protective effect against BP for 3+ months.

#### 3.2.5 Sensitivity analysis and Benjamini–Hochberg corrected test

The results of our MR analysis were subjected to a comprehensive set of sensitivity tests to ensure the robustness and reliability of the findings. We employed the IVW test, MR-Egger’s intercept test, and the MR-PRESSO global test (Supplementary Table 10). Importantly, the vast majority of IVW test, relying on Cochran’s Q test, demonstrated a lack of heterogeneity (*P* > 0.05), indicating consistency across our analyses. Furthermore, neither MR-Egger’s intercept test nor the MR-PRESSO global test detected evidence of horizontal pleiotropy (*P* > 0.05), reinforcing the reliability of our findings. Scatterplots illustrating the outcomes of the five MR analysis methods have been presented in Supplementary Figures 1–8. In addition, a leave-one-out analysis, as illustrated in Supplementary Figures 9–16, demonstrated that the exclusion of any single SNP did not significantly alter the overall results, confirming the stability and reliability of our findings. To control for false positives, we applied the Benjamini–Hochberg corrected test. Corrected for multiple testing, *Oxalobacter* (OR: 1.143, 95% CI 1.061–1.232, *P* = 0.0004) and *Tyzzerella 3* (OR: 1.145, 95% CI 1.059–1.238, *P* = 0.0007) were identified as risk factors for LBP, while *Ruminococcaceae UCG011* (OR: 0.859, 95% CI 0.791–0.932, *P* = 0.0003) was identified as a protective factor against LBP. Additionally, *Ruminococcus gnavus group* (OR: 0.998, 95% CI 0.996–0.999, *P* = 0.0004) was revealed as a protective factor specifically against lumbar and other intervertebral disk disorders with radiculopathy, and *Olsenella* (OR: 0.893, 95% CI 0.839–0.951, *P* = 0.0004) emerged as a protective factor against low back pain or/and sciatica.

## 4 Discussion

To the best of our knowledge, this study stands as the inaugural and thorough investigation employing publicly available GWAS databases to scrutinize the causal relationship between the gut microbiota and four prevalent types of SP—specifically, NP, TSP, LBP, and BP. Through a meticulous analysis of the data, we observed that all IVW results within the dataset for ‘ lumbar and other intervertebral disk disorders with radiculopathy ’ were close to 1, with narrow 95% confidence intervals. This suggests the absence of a significant causal effect between the gut microbiota and this particular type of LBP. Consequently, we opted to exclude the data related to this specific type of LBP. Following a stringent selection process, we successfully identified a total of 37 bacterial genera exhibiting potential causal relationships with SP. Subsequently, through the application of the Benjamini–Hochberg corrected test, we corroborated the strong causal relationship of four bacterial genera with SP. The discoveries emerging from this study serve as an invaluable reference for subsequent research endeavors aimed at preventing and managing SP through precise interventions in the gut microbiota.

The study demonstrates that over 90% of cases involving SP lack a definitive diagnosis related to identifiable causes such as fractures, infections, tumors, etc (Haldeman et al., 2012). Consequently, clinical interventions face challenges in delivering precise and effective targeted treatments, often relying on symptomatic relief measures such as analgesics and soft tissue relaxation (Cheng, 2021). In recent years, growing attention has been directed toward understanding the intricate relationship between the gut microbiota and pain. The gut microbiota, with its array of metabolites, neurotransmitters, by-products, and other mediators, possesses the ability to modulate pain (Guo et al., 2019). In this context, we conducted a comprehensive exploration of the causal relationship between the gut microbiota and SP using Mendelian randomization analysis. Upon subjecting the MR results to the Benjamini–Hochberg corrected test, a strong causal relationship emerged between the gut microbiota and low back pain or/and lower limb pain. However, no significant causal relationship was discerned between the gut microbiota and NP, TSP, or BP. The outcomes of our study align with the observations of Arai et al. (2018), who reported a noteworthy positive relationship between the severity of constipation and the intensity of pain in individuals experiencing low back pain or/and lower limb pain. Notably, the severity of constipation is known to exert a significant influence on the composition of the gut microbiota (Zhu et al., 2014).

We observed that *Oxalobacter* and *Tyzzerella 3* act as risk factors for low back pain or/and lower limb pain, while *Ruminococcaceae UCG011* and *Olsenella* exhibit protective effects. Rajasekaran et al. (2020) identified a higher abundance of *Oxalobacter* in degenerated intervertebral disks. Intervertebral disk degeneration is widely recognized as a crucial factor in the onset and progression of LBP (Meng et al., 2023). Hence, we posit that *Oxalobacter* might contribute to LBP by potentially inducing intervertebral disk degeneration. Currently, there is limited clinical or experimental research on *Tyzzerella 3* concerning LBP, and direct evidence establishing *Tyzzerella 3* as a risk factor is lacking. Some studies found that *Tyzzerella* can produce aromatic amines and is positively correlated with the occurrence of diabetes (Ruuskanen et al., 2022; Sugiyama et al., 2022). Therefore, *Tyzzerella 3* might promote the development of LBP by influencing endocrine or endothelial functions. Regarding the protective bacterial genera identified in the development of LBP, relevant studies on changes in the abundance of *Ruminococcaceae UCG011* and *Olsenella* in the feces of LBP patients or animal models are currently unavailable. *Ruminococcaceae UCG011* belongs to the *Ruminococcaceae* family, and evidence indicates a significant reduction in *Ruminococcaceae* family bacteria in the feces of mice with lumbar disk herniation compared to the control group (Wang et al., 2021). Butyrate produced by *Ruminococcaceae* family bacteria can promote the synthesis of vitamin D in the body (Murdaca et al., 2021). Studies have found that vitamin D has a regulatory effect on the immune function of the body, as it can inhibit the differentiation of pro-inflammatory cells such as T helper (Th) 17 cells, thereby reducing the secretion of pro-inflammatory factors such as IL-17 (Murdaca et al., 2011; Yamamoto and Jørgensen, 2020). Therefore, we hypothesize that improving local chronic inflammation in the body may be a potential mechanism for the protective effect of *Ruminococcaceae UCG011*. Additionally, research indicates that Short-chain fatty acids (SCFAs) produced by *Olsenella* can mediate the immune balance between Th17 cells and regulatory T cells (Tregs) by activating Tregs’ activity, thus balancing the levels of pro-inflammatory factors (derived from Th17 cells) and anti-inflammatory factors (derived from Treg cells) in the body (Kim, 2021). This could be a potential mechanism by which *Olsenella* resists the development of LBP. The involvement of the gut microbiota in the development of LBP is an exceedingly complex process. Therefore, the specific mechanisms by which the four identified positive gut microbiota affect low back pain or/and lower limb pain, require further experimental validation in subsequent studies.

In addition to the four bacterial genera strongly associated with SP previously mentioned, our study identified 33 other bacterial genera exhibiting potential causal relationship with SP. To ensure a comprehensive exploration of gut microbiota potentially linked to SP, we incorporated data from all gut microbiota groups with Benjamini–Hochberg corrected test p-values below 0.05. Among the eight bacterial genera potentially causally related to NP, the majority belonged to the *Firmicutes* phylum. Qin et al. (2019) reported symptom improvement in cervical spondylosis patients following manual therapy, accompanied by a significant increase in the proportions of *Lactobacillus* and *Bifidobacterium* in feces, both belonging to the *Firmicutes* phylum. Unfortunately, limited clinical and foundational research exists on the gut microbiota’s association with NP, and there is no evidence supporting the relationship between *Lachnospiraceae UCG010*, *Escherichia-Shigella*, *Methanobrevibacter*, *Eubacterium nodatum group*, *Faecalibacterium*, *Subdoligranulum*, *Butyrivibrio*, *Ruminococcaceae UCG010*, and NP. Potential protective factors for TSP include *Butyrivibrio*, *Ruminococcaceae UCG011*, and *Eubacterium brachy group*. However, similar to NP, research on the gut microbiota and TSP is scarce, lacking supporting evidence. Interestingly, through a comparative analysis of potential causally related bacterial genera in NP and TSP, *Butyrivibrio* was identified as a shared potential protective genus. Its production of butyrate, propionate, and other SCFAs inhibiting histone deacetylase activity may be one of the potential mechanisms underlying *Butyrivibrio*’s analgesic effects (Descalzi et al., 2015; Tang et al., 2022). Research on the relationship between gut microbiota and low back pain or/and lower limb pain is more abundant. Yao et al. (2023) found a significant decrease in *Lachnospiraceae NK4A136 group*, *Turicibacter*, and *Roseburia* in the feces of rats in a LBP model compared to the control group. In various LBP animal models, Wang et al. (2021) also found lower abundance of *Lachnospiraceae NK4A136 group* in rats with lumbar disk herniation compared to the control group. A cross-sectional study revealed a significant reduction in skeletal muscle mass index in patients with LBP compared to normal individuals (Park et al., 2023). Sugimura et al. (2022) found a positive relationship between the abundance of *Eisenbergiella* and skeletal muscle mass, suggesting a reasonable hypothesis that *Eisenbergiella* may prevent LBP by increasing lumbar skeletal muscle mass. Consistent with the above findings, our study identified *Lachnospiraceae NK4A136 group*, *Turicibacter*, *Roseburia*, and *Eisenbergiella* as potential protective factors for LBP. In studies investigating the relationship between BP and gut microbiota, Dekker Nitert et al. (2020) found a higher abundance of the genus *Uncl. Christensenellaceae* in patients with BP. However, contrary to this conclusion, our results indicate that *Christensenellaceae R.7 group* is a potential protective factor for BP, a finding supported by Hollister et al. (2020). In summary, this study experimentally validated some of the bacterial genera with potential causal relationships to SP that were screened. This underscores the significance of exploring bacterial genera with potential causal relationships to SP, and the unvalidated genera warrant further investigation.

Whether it is NP, TSP, LBP, or BP, they represent distinct segments of SP. Fundamentally, these pains originate in the spinal column, suggesting a certain degree of interconnectedness among pains occurring in different spinal segments. To dissect this phenomenon, we conducted a comparative analysis of bacterial genera potentially influencing SP across different segments. Our investigation revealed that *Ruminococcaceae UCG010* exhibits potential protective properties, common to both NP in the last month and BP in the last month. Similarly, *Ruminococcaceae UCG011* emerges as a potential safeguard against TSP, LBP, and the amalgamation of low back pain or/and sciatica. Notably, *Butyrivibrio* surfaces as a potential protective agent against NP in the last month and TSP. Intriguingly, the *Eubacterium nodatum group* assumes a potential risk factor role in NP for 3+ months while concurrently acting as a potential protective factor for low back pain or/and sciatica. This nuanced outcome suggests that a singular bacterial genus might exert counteractive regulatory influences on pains manifesting in different spinal segments. In summation, the intricate modulation of pains across different spinal segments by the gut microbiota underscores a complex process. Shared or divergent mechanisms may underscore these phenomena, necessitating further exploration to unveil the underlying intricacies.

Furthermore, to scrutinize potential disparities in causally related bacterial genera associated with SP across varying durations, we incorporated datasets reflecting distinct timeframes— 1 month and 3 months. Regrettably, datasets for different durations of TSP and low back pain or/and lower limb pain, were unavailable. Consequently, our analysis encompassed datasets for 1 month and 3 months of NP and BP. MR outcomes reveal an absence of shared potential causally related bacterial genera between 1 month and 3 months of NP, a trend also mirrored in the context of BP. This suggests a dynamic nature, signifying that bacterial genera with potential causal relationship with SP may evolve during the course of its progression. Contrary to the findings of Dekker Nitert et al. (2020), who observed heightened abundance of *Adlercreutzia*, *Roseburia*, and *Uncl. Christensenellaceae* in patients with 1 month of BP, our MR results challenge this observation. Specifically, *Adlercreutzia* (*P* = 0.53) and *Roseburia* (*P* = 0.92) do not emerge as potential causally related bacterial genera for 1 month of BP. Instead, the *Christensenellaceae R.7 group* surfaces as a potential protective bacterial genus for this duration of BP. We posit that discrepancies in results may be attributed to the relatively modest sample size employed in the clinical study. In conclusion, it is imperative to delineate causally related bacterial genera associated with SP at distinct developmental stages. This endeavor holds paramount significance for tailoring precise interventions targeting SP across varying durations.

The robustness of this investigation stems from the application of MR to evaluate the causal relationship between gut microbiota and SP. By leveraging genetic variations as IVs, MR effectively mitigates external confounding factors, thereby bolstering internal validity owing to the inherent randomness and independence of these genetic determinants. Notably, subgroup analyses conducted for distinct durations of spinal pain (i.e., 1 month and 3 months) contribute valuable insights, offering implications for future interventions targeting specific gut microbiota to address SP across varying timeframes. However, our study contends with several limitations. Firstly, the exclusivity of data from European countries prompts the necessity for further experimental validation to ascertain the generalizability of MR results to populations in other regions, such as Asia and Africa. Secondly, the adherence to the conventional significance threshold for SNPs in MR studies (*P* < 5e−08) restricts the number of selected SNPs, potentially influencing subsequent sensitivity analyses and control for horizontal pleiotropy. Thirdly, the constraint of exposure data to the genus level hampers a more nuanced analysis of the causal relationship between higher taxonomic levels of gut microbiota and SP. Lastly, relying on public databases rather than original sources limits the scope of available datasets, resulting in the omission of subgroup analyses for TSP and low back pain or/and lower limb pain during SP subgroup analyses— a notable constraint in our study.

## 5 Conclusion

Through our two-sample Mendelian randomization (MR) analysis, we have successfully identified a comprehensive set of 37 bacterial genera potentially causally related to SP, encompassing NP, TSP, LBP, and BP. Employing the rigorous Benjamini-Hochberg corrected test, we have discerned four bacterial genera that exhibit robust causal relationships with SP. Notably, our analysis has unveiled a potential common regulatory mechanism, suggesting that certain bacterial genera may influence spinal pain across different segments. Furthermore, the dynamic nature of these potential causal relationships has been illuminated, indicating that, as the duration of SP progresses, the involved bacterial genera undergo changes. In conclusion, this study contributes valuable insights into the intricate interplay between the gut microbiota and SP. Such revelations not only serve as a significant reference for understanding these complex connections but also pave the way for future research endeavors aimed at preventing and treating SP through targeted interventions in the gut microbiota.

## Data availability statement

The original contributions presented in the study are included in the article/Supplementary material, further inquiries can be directed to the corresponding author/s.

## Author contributions

SH: Conceptualization, Writing – original draft. LC: Conceptualization, Writing – original draft. XZ: Formal analysis, Writing – review and editing. YH: Data curation, Writing – review and editing. YT: Writing – review and editing. HH: Writing – review and editing. HW: Validation, Writing – review and editing. CY: Validation, Writing – review and editing. ZL: Funding acquisition, Project administration, Writing – review and editing. LL: Funding acquisition, Validation, Writing – review and editing. BY: Writing – review and editing.

## Footnotes

1
https://gwas.mrcieu.ac.uk/

## Supplementary material

Supplementary Data Sheet 1 Genetically predicted causal effects of gut microbiota on spinal pain: a two-sample Mendelian Randomization analysis.

Supplementary Data Sheet 2 Scatterplot results for five analysis methods and leave-one-out analysis results.

Supplementary Data Sheet 3 Analysis codes for MR analysis.

## References

[B1] Akagawa S. Kaneko K. (2022). Gut microbiota and allergic diseases in children. *Allergol. Int.* 3 301–309. 10.1016/j.alit.2022.02.004 35314107

[B2] Arai Y. Shiro Y. Funak Y. Kasugaii K. Omichi Y. Sakurai H. (2018). The association between constipation or stool consistency and pain severity in patients with chronic pain. *Anesth. Pain Med.* 4:e69275. 10.5812/aapm.69275 30250817 PMC6139698

[B3] Bowden J. Davey Smith G. Burgess S. (2015). Mendelian randomization with invalid instruments: effect estimation and bias detection through Egger regression. *Int. J. Epidemiol.* 2 512–525. 10.1093/ije/dyv080 26050253 PMC4469799

[B4] Bowden J. Davey Smith G. Haycock P. Burgess S. (2016). Consistent estimation in mendelian randomization with some invalid instruments using a weighted median estimator. *Genet. Epidemiol.* 4 304–314. 10.1002/gepi.21965 27061298 PMC4849733

[B5] Burgess S. Bowden J. Fall T. Ingelsson E. Thompson S. (2017). Sensitivity analyses for robust causal inference from mendelian randomization analyses with multiple genetic variants. *Epidemiology* 1 30–42. 10.1097/EDE.0000000000000559 27749700 PMC5133381

[B6] Burgess S. Dudbridge F. Thompson S. (2016). Combining information on multiple instrumental variables in mendelian randomization: comparison of allele score and summarized data methods. *Stat. Med.* 11 1880–1906. 10.1002/sim.6835 26661904 PMC4832315

[B7] Burgess S. Thompson S. (2011). Bias in causal estimates from Mendelian randomization studies with weak instruments. *Stat. Med.* 11 1312–1323. 10.1002/sim.4197 21432888

[B8] Chassaing B. Etienne-Mesmin L. Gewirtz A. (2014). Microbiota-liver axis in hepatic disease. *Hepatology* 1 328–339. 10.1002/hep.26494 23703735 PMC4084781

[B9] Cheng Z. (2021). The treatment strategies of spinal pain. *Chin. J. Painol.* 4 339–341. 10.3760/cma.j.cn101658-20210719-00101 30704229

[B10] Clark J. Coopersmith C. (2007). Intestinal crosstalk: a new paradigm for understanding the gut as the “motor” of critical illness. *Shock* 4 384–393. 10.1097/shk.0b013e31805569df 17577136 PMC2084394

[B11] Davies N. Holmes M. Davey Smith G. (2018). Reading Mendelian randomisation studies: a guide, glossary, and checklist for clinicians. *BMJ* 362:k601. 10.1136/bmj.k601 30002074 PMC6041728

[B12] de Luca K. Tavares P. Yang H. Hurwitz E. Green B. Dale H. (2023). Spinal pain, chronic health conditions and health behaviors: data from the 2016-2018 national health interview survey. *Int. J. Environ. Res. Public Health* 7:5369. 10.3390/ijerph20075369 37047983 PMC10094294

[B13] Dekker Nitert M. Mousa A. Barrett H. Naderpoor N. de Courten B. (2020). Altered gut microbiota composition is associated with back pain in overweight and obese individuals. *Front. Endocrinol.* 11:605. 10.3389/fendo.2020.00605 32982987 PMC7492308

[B14] Descalzi G. Ikegami D. Ushijima T. Nestler E. Zachariou V. Narita M. (2015). Epigenetic mechanisms of chronic pain. *Trends Neurosci.* 4 237–246. 10.1016/j.tins.2015.02.001 25765319 PMC4459752

[B15] Emdin C. Khera A. Kathiresan S. (2017). Mendelian Randomization. *JAMA* 19 1925–1926. 10.1001/jama.2017.17219 29164242

[B16] GBD 2015 Disease and Injury Incidence and Prevalence Collaborators (2016). Global, regional, and national incidence, prevalence, and years lived with disability for 310 diseases and injuries, 1990-2015: a systematic analysis for the Global Burden of Disease Study 2015. *Lancet* 10053 1545–1602. 10.1016/S0140-6736(16)31678-6 27733282 PMC5055577

[B17] Greco M. Minelli C. Sheehan N. Thompson J. (2015). Detecting pleiotropy in Mendelian randomisation studies with summary data and a continuous outcome. *Stat. Med.* 21 2926–2940. 10.1002/sim.6522 25950993

[B18] Guo R. Chen L. Xing C. Liu T. (2019). Pain regulation by gut microbiota: molecular mechanisms and therapeutic potential. *Br. J. Anaesth.* 5 637–654. 10.1016/j.bja.2019.07.026 31551115

[B19] Haldeman S. Kopansky-Giles D. Hurwitz E. Hoy D. Mark Erwin W. Dagenais S. (2012). Advancements in the management of spine disorders. *Best Pract. Res. Clin. Rheumatol.* 2 263–280. 10.1016/j.berh.2012.03.006 22794098

[B20] Hollister E. Cain K. Shulman R. Jarrett M. Burr R. Ko C. (2020). Relationships of microbiome markers with extraintestinal, psychological distress and gastrointestinal symptoms, and quality of life in women with irritable bowel syndrome. *J. ClinGastroenterol.* 2 175–183. 10.1097/MCG.0000000000001107 30148765 PMC6387862

[B21] Jandhyala S. Talukdar R. Subramanyam C. Vuyyuru H. Sasikala M. Nageshwar Reddy D. (2015). Role of the normal gut microbiota. *World J Gastroenterol.* 29 8787–8803. 10.3748/wjg.v21.i29.8787 26269668 PMC4528021

[B22] Kim C. (2021). Control of lymphocyte functions by gut microbiota-derived short-chain fatty acids. *Cell Mol. Immunol.* 5 1161–1171. 10.1038/s41423-020-00625-0 33850311 PMC8093302

[B23] Koes B. van Tulder M. Thomas S. (2006). Diagnosis and treatment of low back pain. *BMJ* 7555 1430–1434. 10.1136/bmj.332.7555.1430 16777886 PMC1479671

[B24] Kurilshikov A. Medina-Gomez C. Bacigalupe R. (2021). Large-scale association analyses identify host factors influencing human gut microbiome composition. *Nat Genet.* 2 156–165. 10.1038/s41588-020-00763-1 33462485 PMC8515199

[B25] Lei T. He Y. Xiao Y. (2015). Application research of Miao medicine liquid dispensing the drug stick therapy in spinal pain. *Chin. J. Biochem. Pharmaceutics* 2 113–115.

[B26] Ley R. Peterson D. Gordon J. (2006). Ecological and evolutionary forces shaping microbial diversity in the human intestine. *Cell* 4 837–848. 10.1016/j.cell.2006.02.017 16497592

[B27] Lin K. Zhu L. Yang L. (2020). Gut and obesity/metabolic disease: focus on microbiota metabolites. *MedComm* 3:e171. 10.1002/mco2.171 36092861 PMC9437302

[B28] Manchikanti L. Boswell M. Singh V. Pampati V. Damron K. Beyer C. (2004). Prevalence of facet joint pain in chronic spinal pain of cervical, thoracic, and lumbar regions. *BMC Musculoskelet Disord.* 5:15. 10.1186/1471-2474-5-15 15169547 PMC441387

[B29] Meng Q. Liu K. Liu Z. Liu J. Tian Z. Qin S. (2023). Digoxin protects against intervertebral disc degeneration via TNF/NF-κB and LRP4 signaling. *Front. Immunol.* 14:1251517. 10.3389/fimmu.2023.1251517 37790932 PMC10544936

[B30] Murdaca G. Colombo B. Puppo F. (2011). The role of Th17 lymphocytes in the autoimmune and chronic inflammatory diseases. *Intern. Emerg. Med.* 6 487–495. 10.1007/s11739-011-0517-7 21258875

[B31] Murdaca G. Gerosa A. Paladin F. Petrocchi L. Banchero S. Gangemi S. (2021). Vitamin D and microbiota: is there a link with allergies? *Int. J. Mol. Sci.* 8:4288. 10.3390/ijms22084288 33924232 PMC8074777

[B32] Park M. Park S. Chung S. (2023). Relationships between skeletal muscle mass, lumbar lordosis, and chronic low back pain in the elderly. *Neurospine* 3 959–968. 10.14245/ns.2346494.247 37798990 PMC10562244

[B33] Qin Y. (2022). Pueraria lobata targeted preparation improves the clinical symptoms of cervical spondylosis by regulating the balance of gut microbiota. *Comput. Math Methods Med.* 2022:2136807. 10.1155/2022/2136807 35126618 PMC8813225

[B34] Qin Y. Wu Y. Lin F. Yang X. Hao F. (2019). Clinical study on the therapy of rubbing abdomen and pinching along the spine for cervical spondylosis of vertebral artery type. *J. New Chin. Med.* 1 217–220. 10.13457/j.cnki.jncm.2019.01.057

[B35] Quaglio A. Grillo T. De Oliveira E. Di Stasi L. Sassaki L. (2022). Gut microbiota, inflammatory bowel disease and colorectal cancer. *World J. Gastroenterol.* 30 4053–4060. 10.3748/wjg.v28.i30.4053 36157114 PMC9403435

[B36] Rajasekaran S. Soundararajan D. Tangavel C. Muthurajan R. Sri Vijay, Anand K. (2020). Human intervertebral discs harbour a unique microbiome and dysbiosis determines health and disease. *Eur. Spine J.* 7 1621–1640. 10.1007/s00586-020-06446-z 32409889

[B37] Ruuskanen M. Erawijantari P. Havulinna A. Liu Y. Méric G. Tuomilehto J. (2022). Gut microbiome composition is predictive of incident type 2 diabetes in a population cohort of 5,572 finnish adults. *Diabetes Care* 4 811–818. 10.2337/dc21-2358 35100347 PMC9016732

[B38] Slob E. Burgess S. (2020). A comparison of robust Mendelian randomization methods using summary data. *Genet. Epidemiol.* 4 313–329. 10.1002/gepi.22295 32249995 PMC7317850

[B39] Smith G. Ebrahim S. (2003). ‘Mendelian randomization’: can genetic epidemiology contribute to understanding environmental determinants of disease? *Int. J. Epidemiol.* 1 1–22. 10.1093/ije/dyg070 12689998

[B40] Soremekun O. Slob E. Burgess S. Romuald Boua P. Fatumo S. Gill D. (2022). Genetically predicted lipid traits, diabetes liability, and carotid intima-media thickness in african ancestry individuals: a mendelian randomization study. *Circ. Genom. Precis. Med.* 6:e003910. 10.1161/CIRCGEN.122.003910 36355609 PMC7613981

[B41] Sugimura Y. Kanda A. Sawada K. Wai K. Tanabu A. Ozato N. (2022). Association between gut microbiota and body composition in japanese general population: a focus on gut microbiota and skeletal muscle. *Int. J. Environ. Res. Public Health* 12:7464. 10.3390/ijerph19127464 35742712 PMC9224415

[B42] Sugiyama Y. Mori Y. Nara M. Kotani Y. Nagai E. Kawada H. (2022). Gut bacterial aromatic amine production: aromatic amino acid decarboxylase and its effects on peripheral serotonin production. *Gut Microbes* 1:2128605. 10.1080/19490976.2022.2128605 36217238 PMC9553188

[B43] Tang Y. Du J. Wu H. Wang M. Liu S. Tao F. (2022). Potential therapeutic effects of short-chain fatty acids on chronic pain. *Curr. Neuropharmacol.* 22 191–203. 10.2174/1570159X20666220927092016 36173071 PMC10788890

[B44] Wang Z. Wu H. Chen Y. Chen H. Wang X. Yuan W. (2021). Lactobacillus paracasei S16 alleviates lumbar disc herniation by modulating inflammation response and gut microbiota. *Front. Nutr.* 8:701644. 10.3389/fnut.2021.701644 34447774 PMC8382687

[B45] Xie L. Zhao H. Chen W. (2023). Relationship between gut microbiota and thyroid function: a two-sample Mendelian randomization study. *Front. Endocrinol.* 14:1240752. 10.3389/fendo.2023.1240752 37822602 PMC10562735

[B46] Yamamoto E. Jørgensen T. (2020). Relationships between vitamin D, gut microbiome, and systemic autoimmunity. *Front. Immunol.* 10:3141. 10.3389/fimmu.2019.03141 32038645 PMC6985452

[B47] Yang R. Chen Z. Cai J. (2023). Fecal microbiota transplantation: emerging applications in autoimmune diseases. *J. Autoimmun.* 141:103038. 10.1016/j.jaut.2023.103038 37117118

[B48] Yao B. Cai Y. Wang W. Deng J. Zhao L. Han Z. (2023). The effect of gut microbiota on the progression of intervertebral disc degeneration. *Orthop. Surg.* 3 858–867. 10.1111/os.13626 36600636 PMC9977585

[B49] Zhang Y. Li S. Gan R. Zhou T. Xu D. Li H. (2015). Impacts of gut bacteria on human health and diseases. *Int. J. Mol. Sci.* 4 7493–7519. 10.3390/ijms16047493 25849657 PMC4425030

[B50] Zhu L. Liu W. Alkhouri R. Baker R. Bard J. Quigley E. (2014). Structural changes in the gut microbiome of constipated patients. *Physiol. Genomics* 18 679–686. 10.1152/physiolgenomics.00082.2014 25073603

