# Supplementary material for: Genetically predicted causal effects of gut microbiota on spinal pain: a two-sample Mendelian randomization analysis
Source: Front Microbiol. 2024 Mar 25;15:1357303. doi: 10.3389/fmicb.2024.1357303 (PMC10999687; doi:10.3389/fmicb.2024.1357303)
Supplement: Supplementary Data Sheet 2 — Scatterplot results for five analysis methods and leave-one-out analysis results. [file Data_Sheet_2.docx]

**Supplementary Figure S1**

Using a comprehensive assessment of the causal impact of exposure (gut microbiota) on the outcome (neck/shoulder pain in the last month) using five distinct Mendelian randomization (MR) analysis methods. The slopes of the lines in the figures represent the specific effects of the exposure on the outcome. (A): Butyrivibrio; (B): Ruminococcaceae UCG010.

ukb-b-18596: Neck/shoulder pain in the last month.


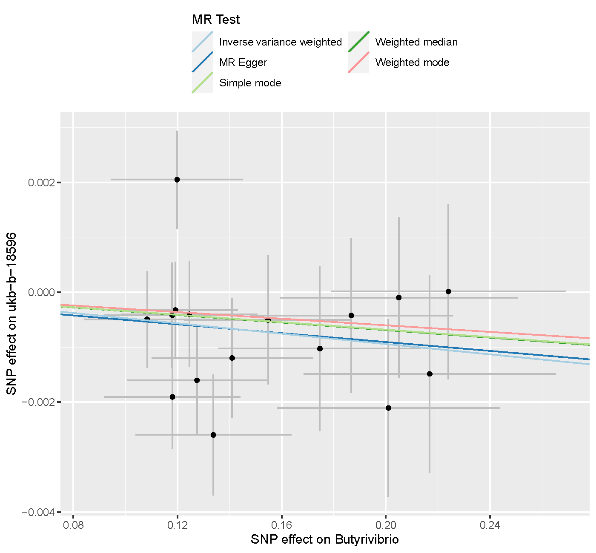

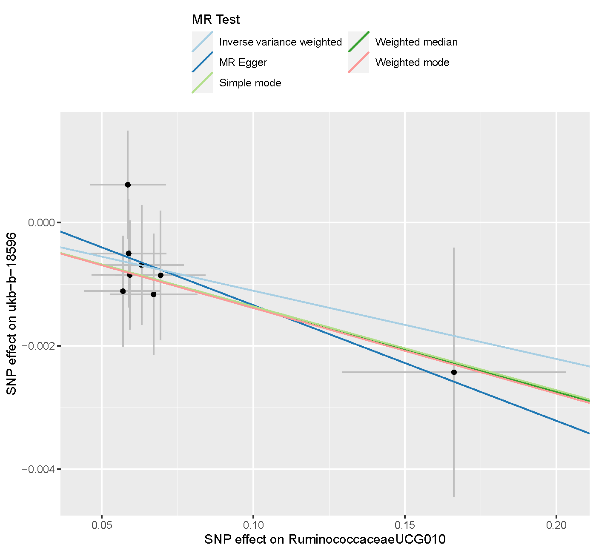


A B

**Supplementary Figure S2**

Using a comprehensive assessment of the causal impact of exposure (gut microbiota) on the outcome (Neck/shoulder pain for 3+ months) using five distinct Mendelian randomization (MR) analysis methods. The slopes of the lines in the figures represent the specific effects of the exposure on the outcome. (A): Lachnospiraceae UCG010; (B): Escherichia-Shigella; (C): Methanobrevibacter; (D): Eubacterium nodatum group; (E): Faecalibacterium; (F): Subdoligranulum.

ukb-b-16118: Neck/shoulder pain for 3+ months


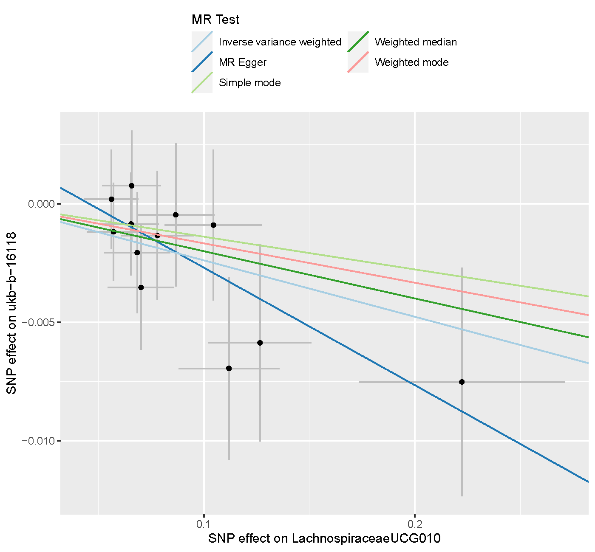

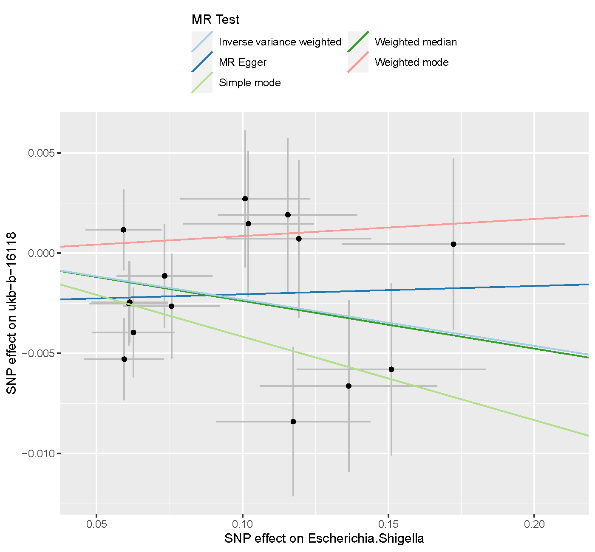


A B


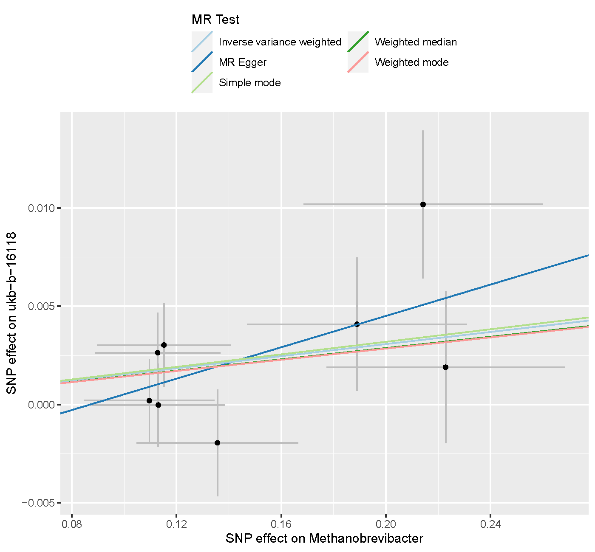

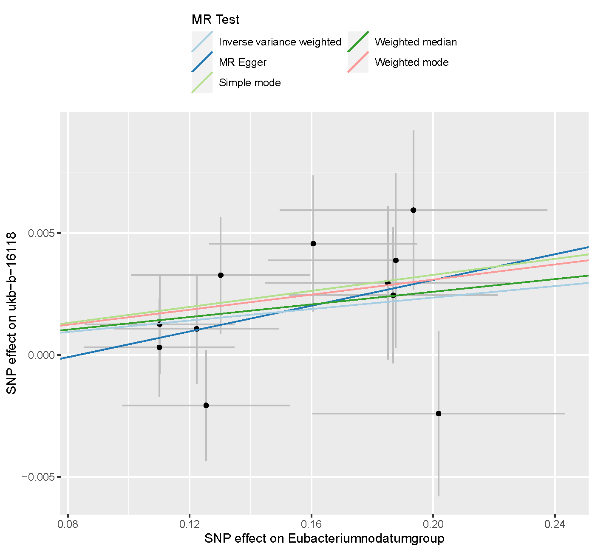


C D


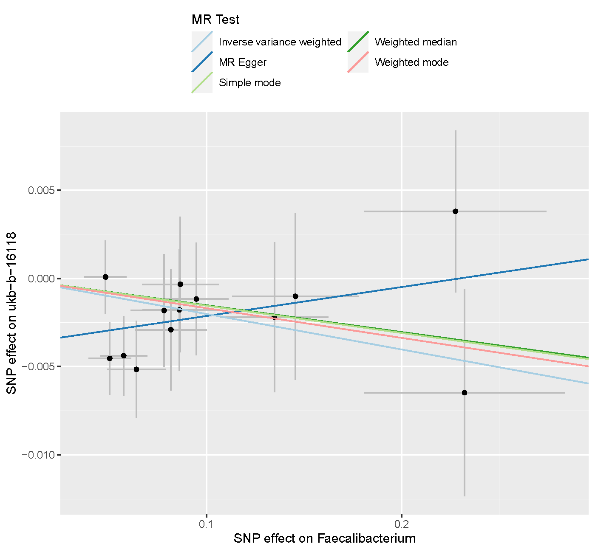

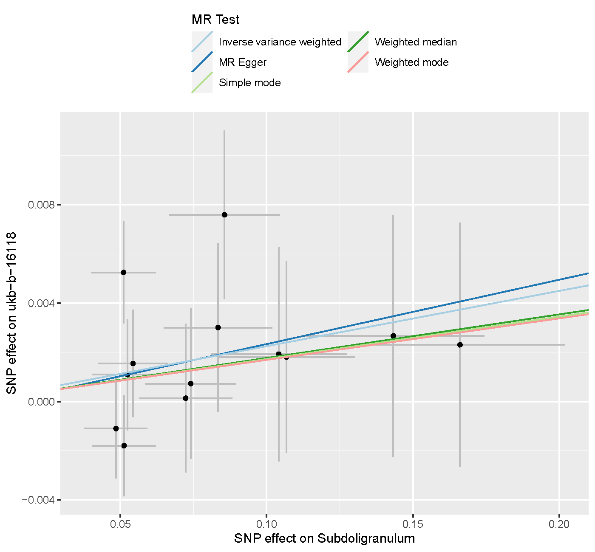


E F

**Supplementary Figure S3**

Using a comprehensive assessment of the causal impact of exposure (gut microbiota) on the outcome (Thoracic spine pain) using five distinct Mendelian randomization (MR) analysis methods. The slopes of the lines in the figures represent the specific effects of the exposure on the outcome. (A): Butyrivibrio; (B): Ruminococcaceae UCG011; (C): Eubacterium brachy group.


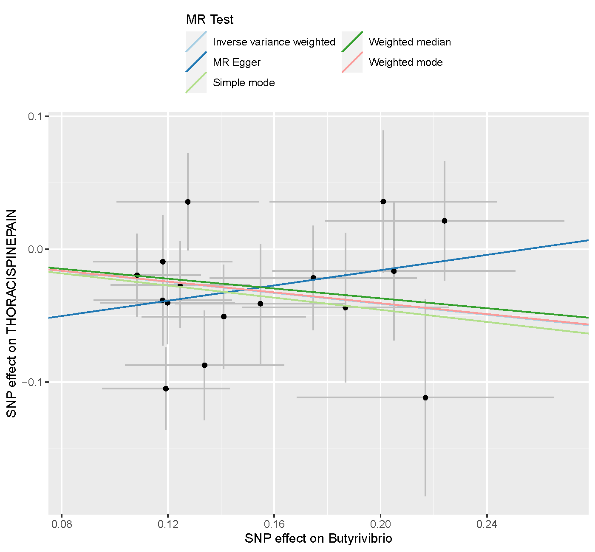

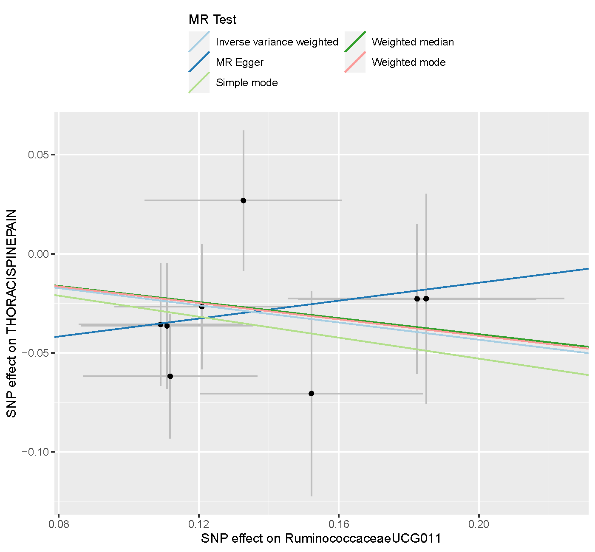


A B


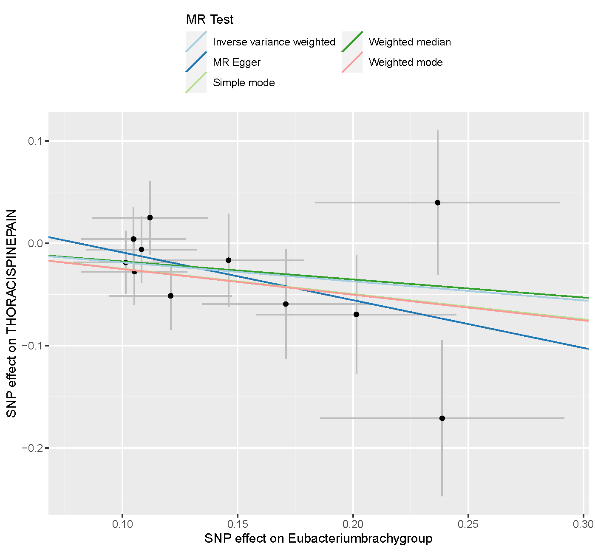


C

**Supplementary Figure S4**

Using a comprehensive assessment of the causal impact of exposure (gut microbiota) on the outcome (Low back pain) using five distinct Mendelian randomization (MR) analysis methods. The slopes of the lines in the figures represent the specific effects of the exposure on the outcome. (A): Ruminococcaceae UCG011; (B): Oxalobacter; (C): Tyzzerella 3; (D): Olsenella; (E): Eisenbergiella; (F): Roseburia.


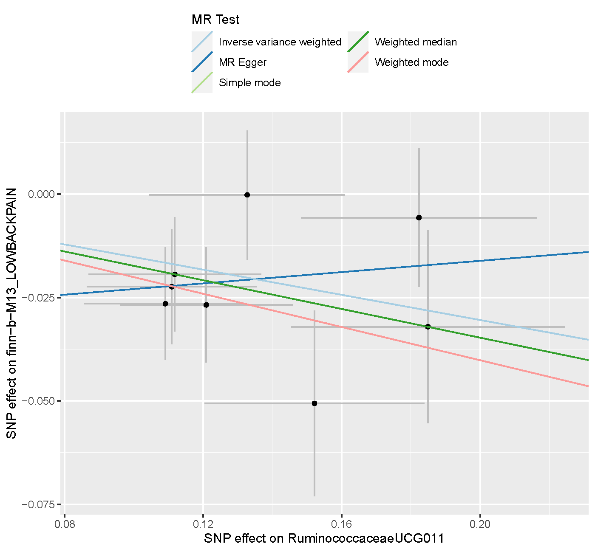

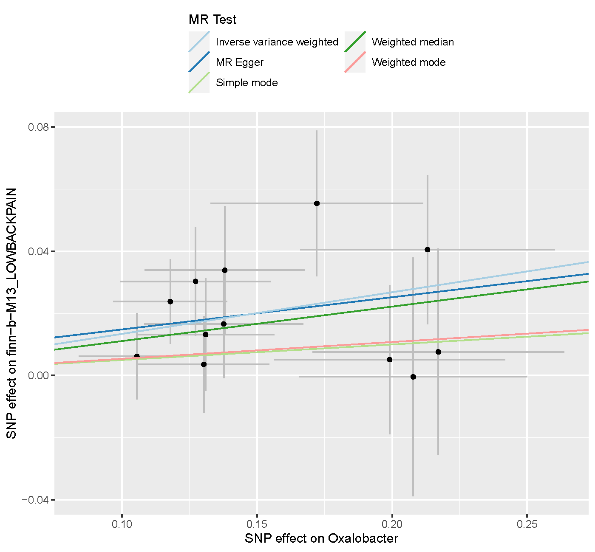


A B


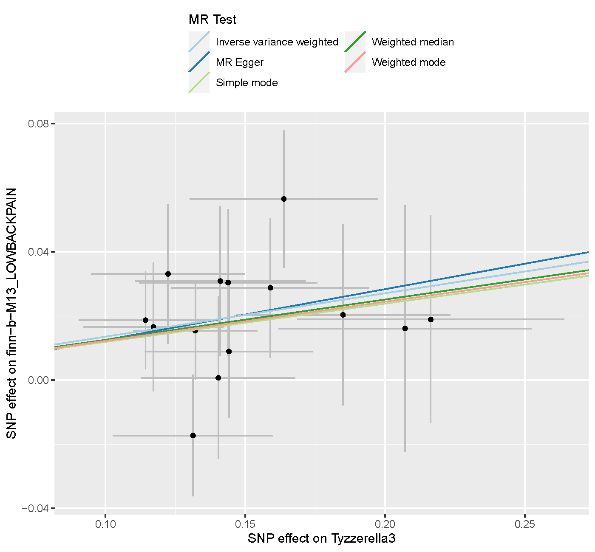

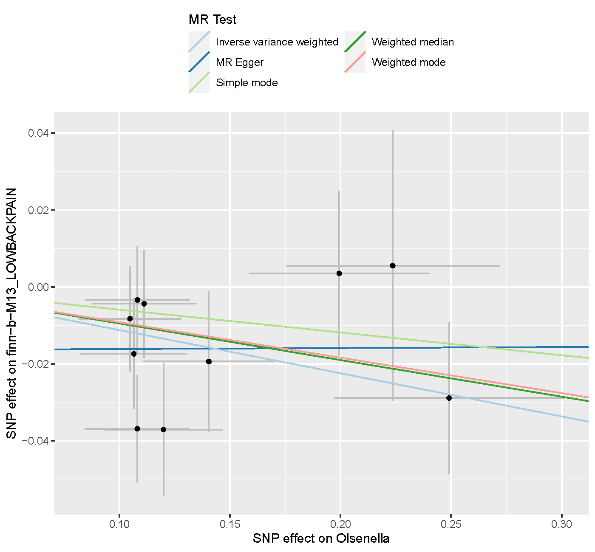


C D


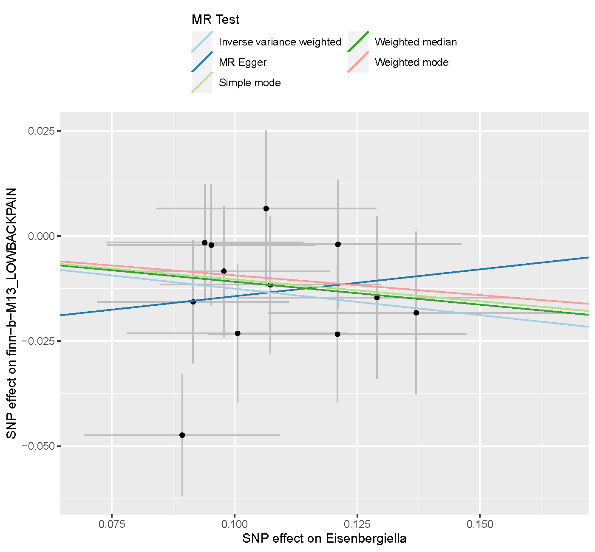

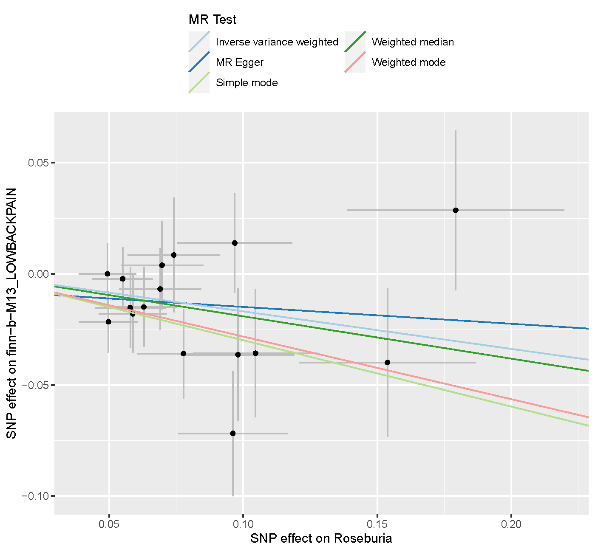


E F

**Supplementary Figure S5**

Using a comprehensive assessment of the causal impact of exposure (gut microbiota) on the outcome (Lumbar and other intervertebral disk disorders with radiculopathy) using five distinct Mendelian randomization (MR) analysis methods. The slopes of the lines in the figures represent the specific effects of the exposure on the outcome. (A): Ruminococcus gnavus group; (B): Eubacterium hallii group; (C): Rikenellaceae RC9 gut group; (D): Prevotella 7; (E): Lachnoclostridium; (F): Oscillibacter; (G): Eubacterium oxidoreducens group.

ukb-b-18279: Lumbar and other intervertebral disk disorders with radiculopathy


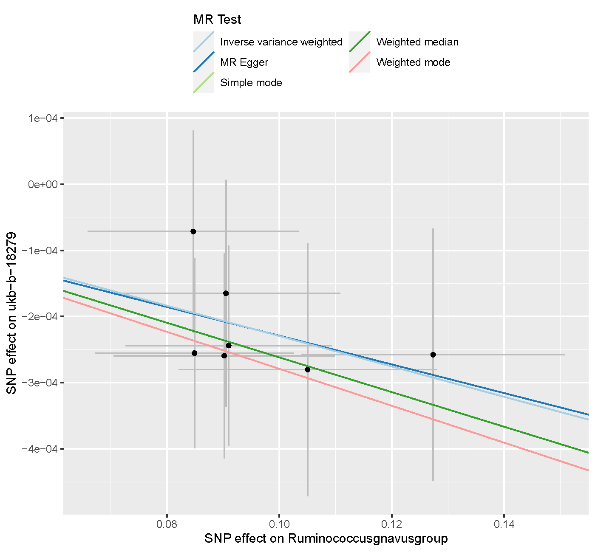

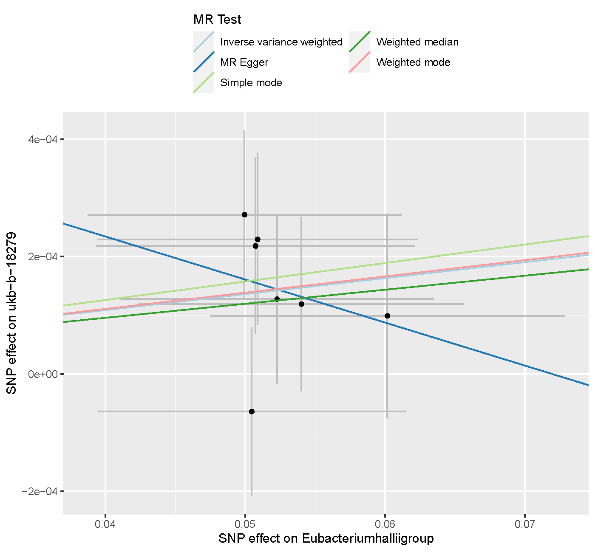


A B


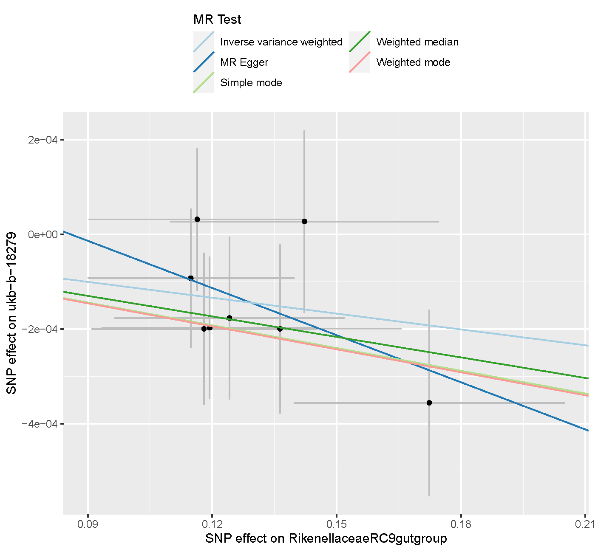

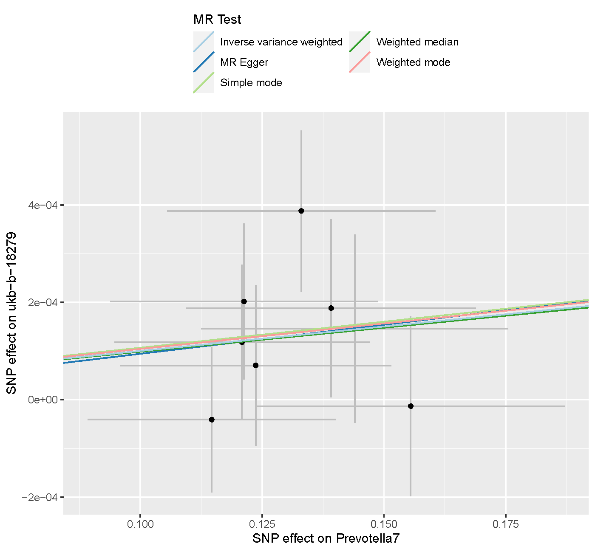


C D


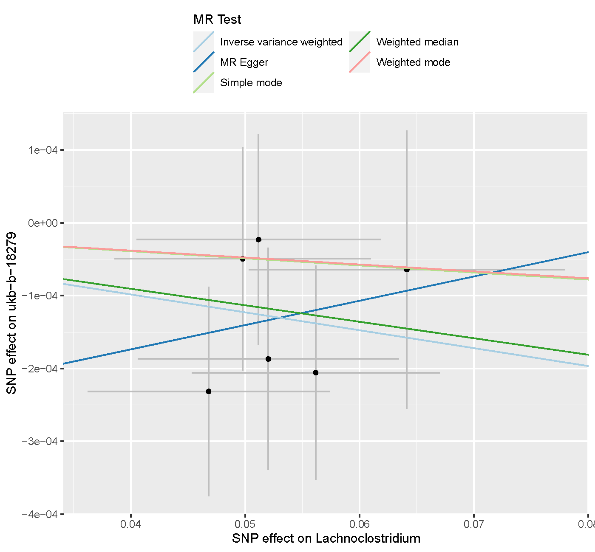

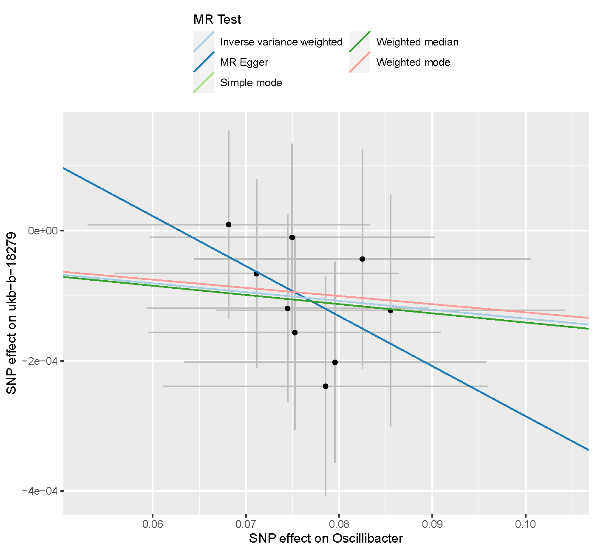


E F


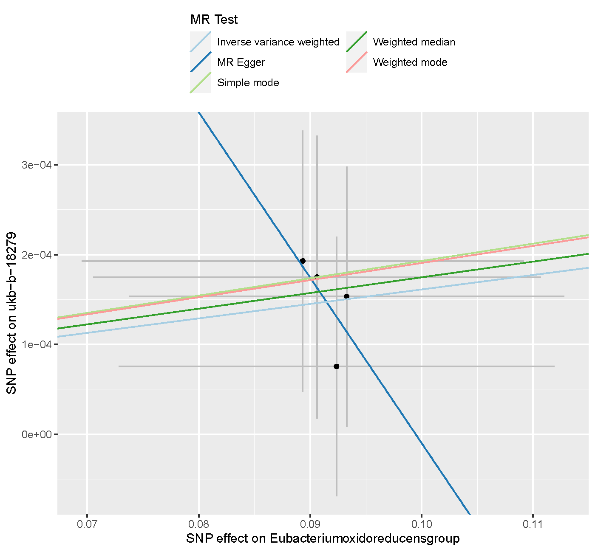


G

**Supplementary Figure S6**

Using a comprehensive assessment of the causal impact of exposure (gut microbiota) on the outcome (Lower back pain or/and sciatica) using five distinct Mendelian randomization (MR) analysis methods. The slopes of the lines in the figures represent the specific effects of the exposure on the outcome. (A): Olsenella; (B): Ruminococcaceae UCG011; (C): Tyzzerella 3; (D): Eisenbergiella; (E): Oxalobacter; (F): Eubacterium fissicatena group; (G): Turicibacter; (H): Eubacterium nodatum group; (I): Lachnospiraceae NK4A136 group; (J): Ruminiclostridium 5; (K): Oscillibacter.


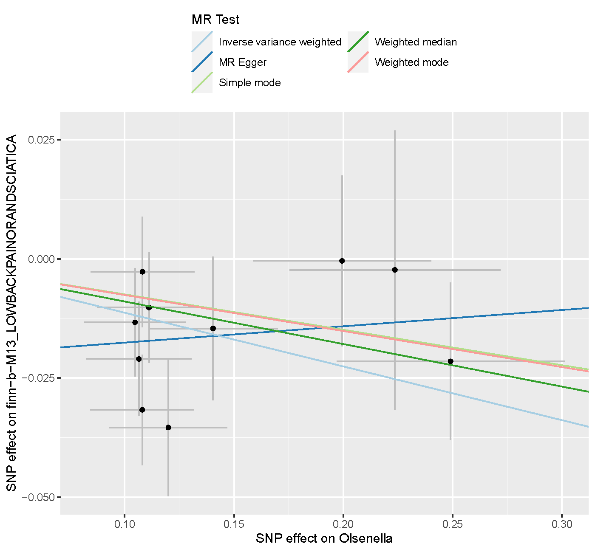

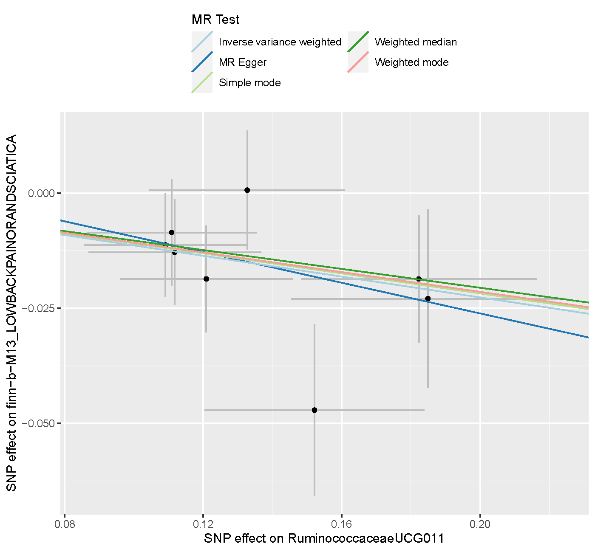


A B


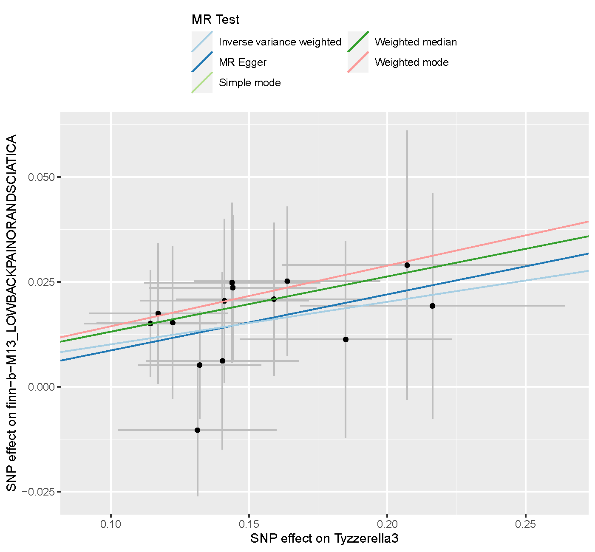

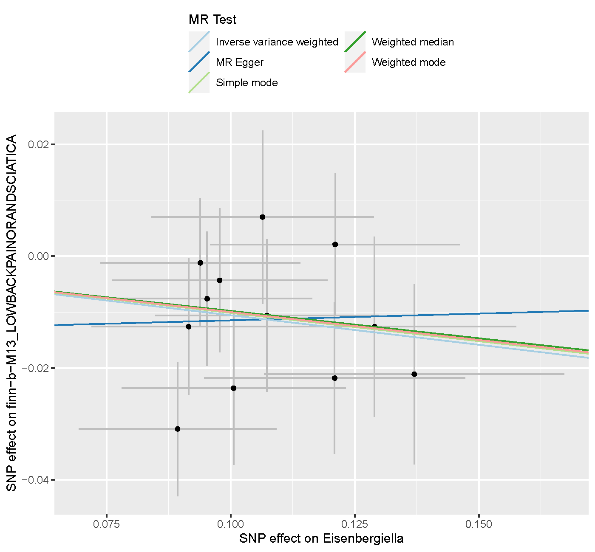


C D


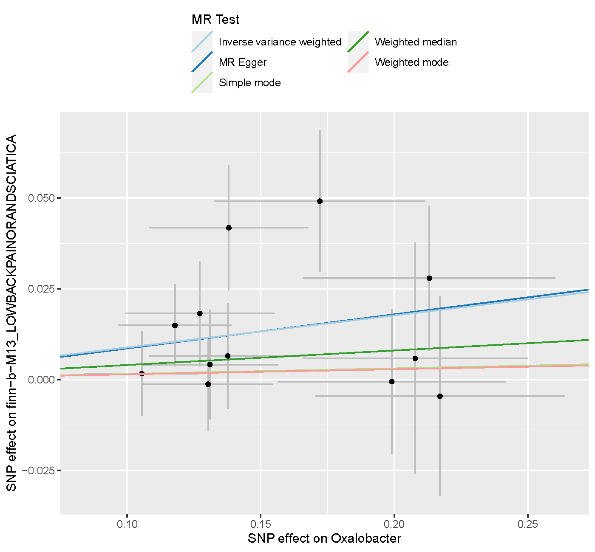

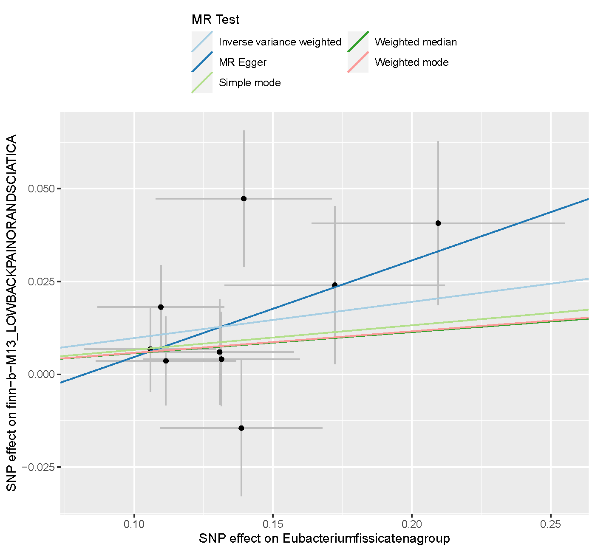


E F


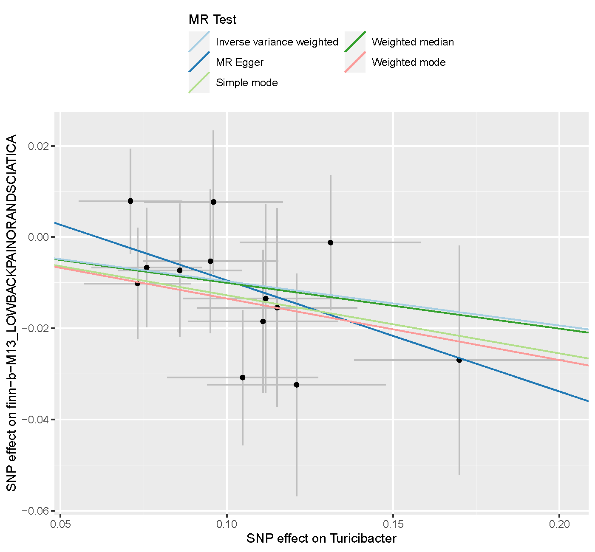

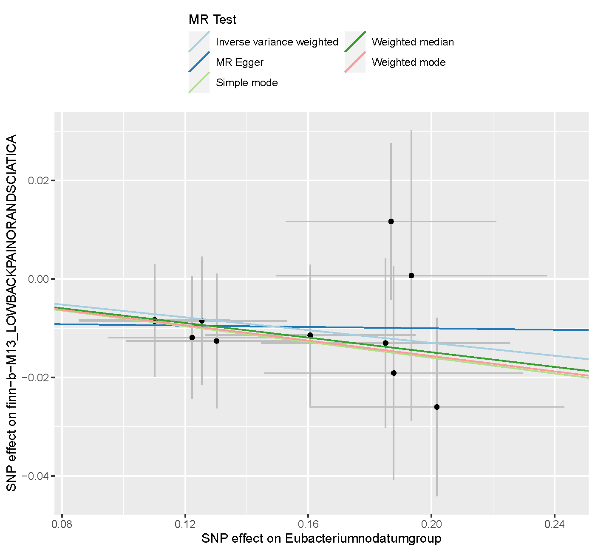


G H


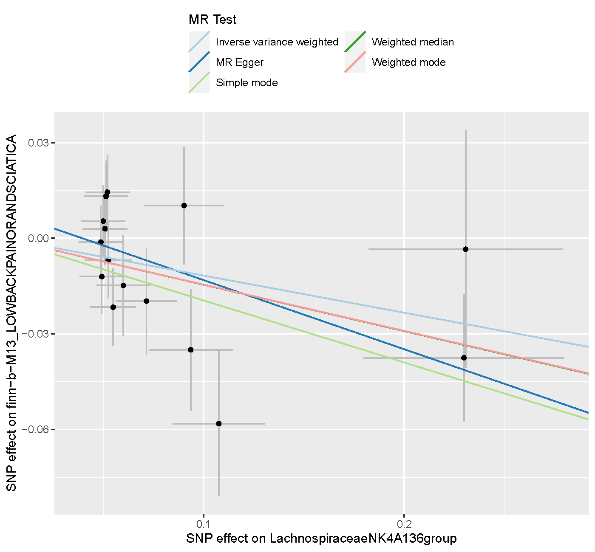

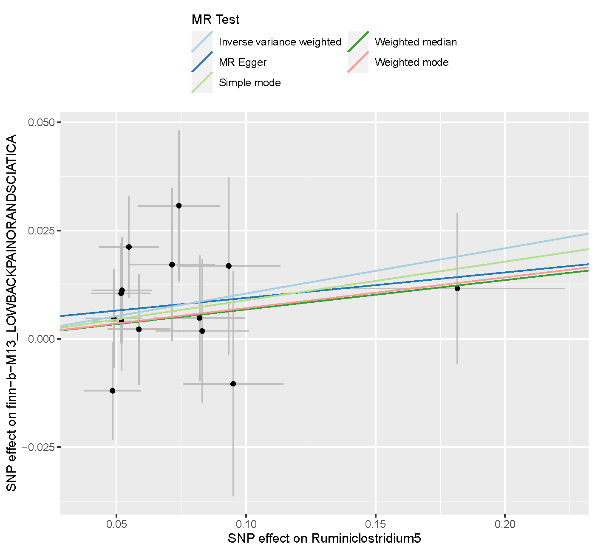


I J


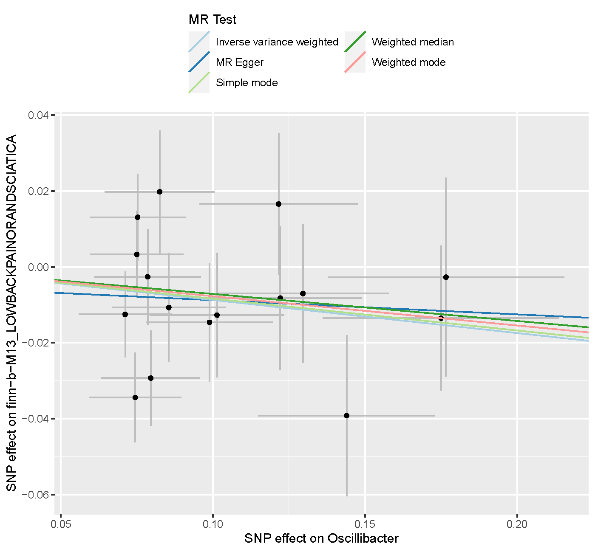


K

**Supplementary Figure S7**

Using a comprehensive assessment of the causal impact of exposure (gut microbiota) on the outcome (Back pain in the last month) using five distinct Mendelian randomization (MR) analysis methods. The slopes of the lines in the figures represent the specific effects of the exposure on the outcome. (A): Alloprevotella; (B): Christensenellaceae R.7 group; (C): Intestinibacter; (D): Eubacterium hallii group; (E): Lachnoclostridium; (F): Oscillospira; (G): Ruminococcaceae UCG010.

ukb-b-9838: Back pain in the last month


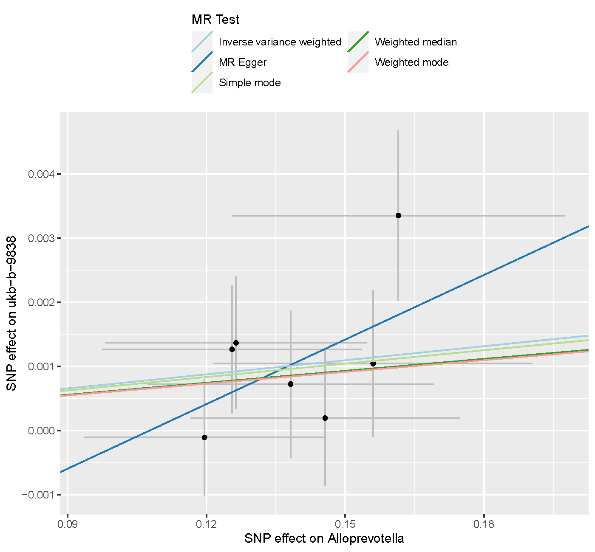

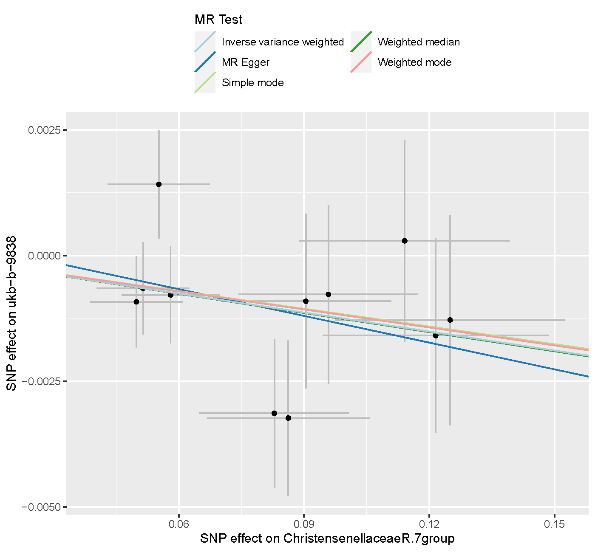


A B


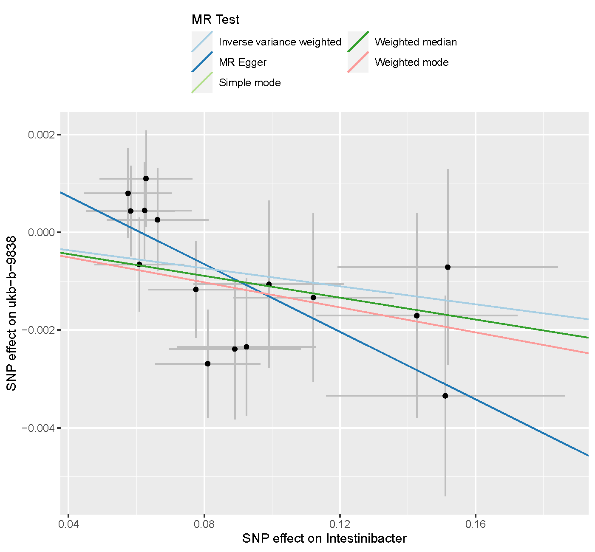

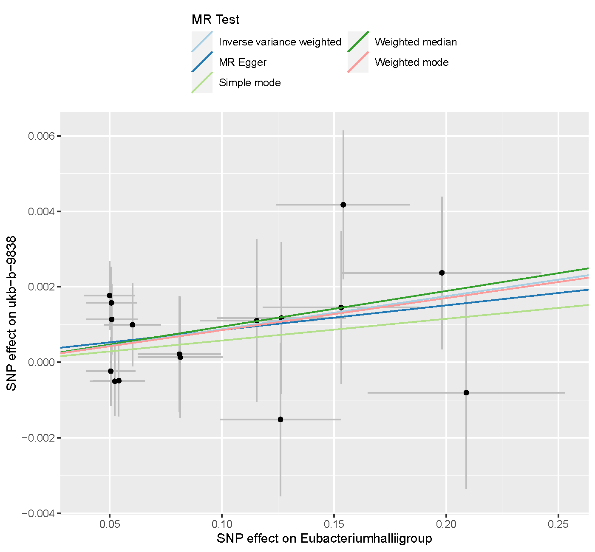


C D


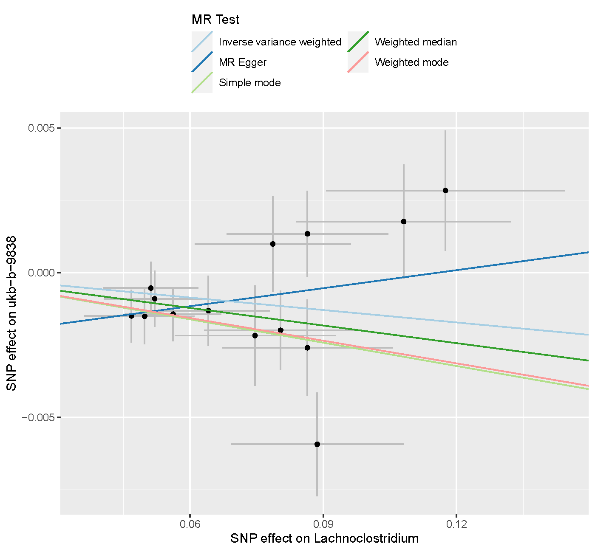

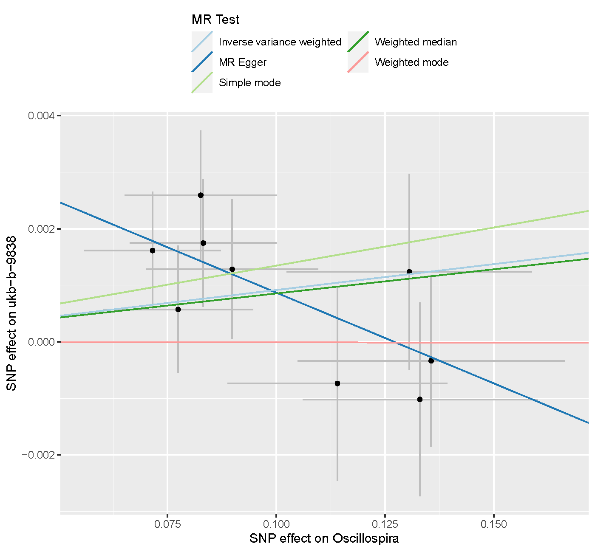


E F


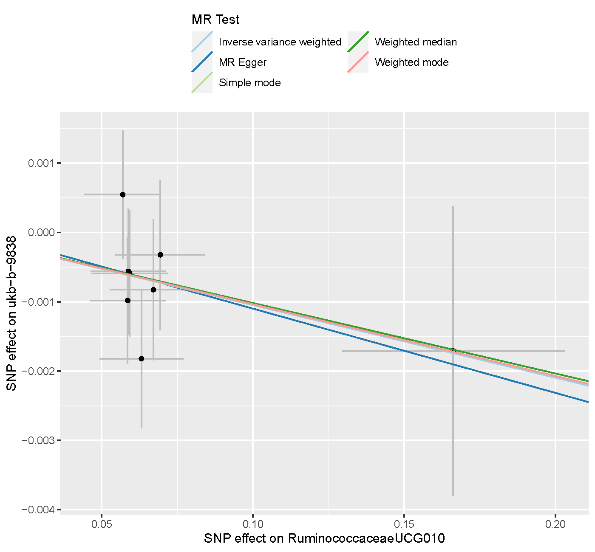


G

**Supplementary Figure S8**

Using a comprehensive assessment of the causal impact of exposure (gut microbiota) on the outcome (Back pain for 3+ months) using five distinct Mendelian randomization (MR) analysis methods. The slopes of the lines in the figures represent the specific effects of the exposure on the outcome. (A): Coprococcus 2; (B): Ruminococcus 1.

ukb-b-8463: Back pain for 3+ months


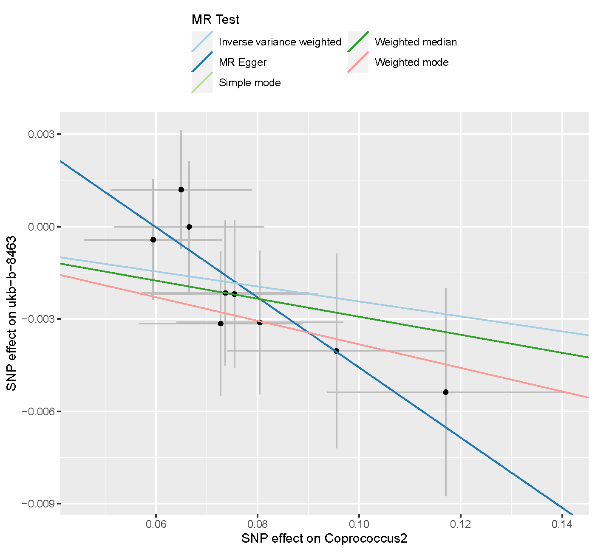

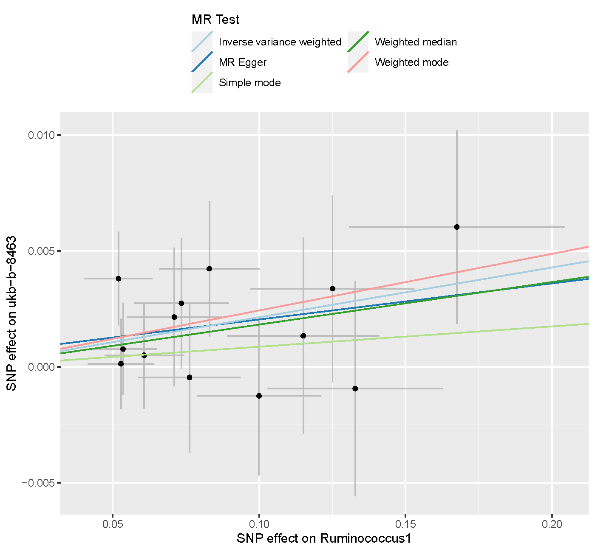


A B

**Supplementary Figure S9**

The stability of Mendelian Randomization (MR) results between exposure (gut microbiota) and the outcome (Neck/shoulder pain in the last month) was assessed using the leave-one-out method. (A): Butyrivibrio; (B): Ruminococcaceae UCG010.

ukb-b-18596: Neck/shoulder pain in the last month.


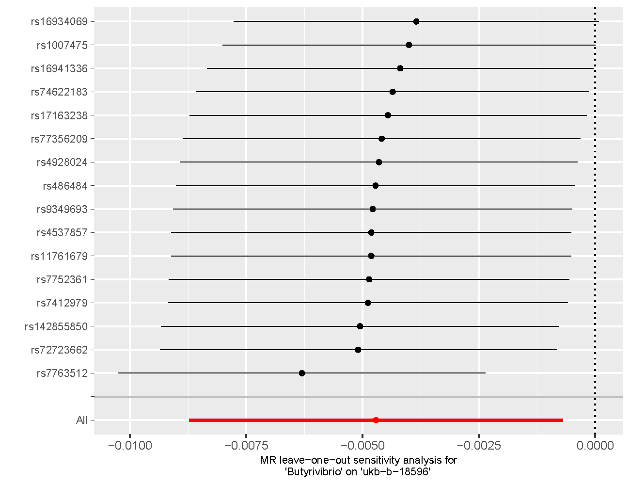

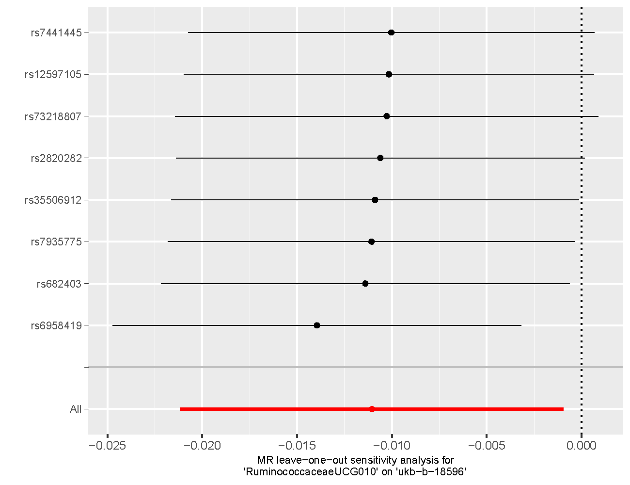


A B

**Supplementary Figure S10**

The stability of Mendelian Randomization (MR) results between exposure (gut microbiota) and the outcome (Neck/shoulder pain for 3+ months) was assessed using the leave-one-out method. (A): Lachnospiraceae UCG010; (B): Escherichia-Shigella; (C): Methanobrevibacter; (D): Eubacterium nodatum group; (E): Faecalibacterium; (F): Subdoligranulum.

ukb-b-16118: Neck/shoulder pain for 3+ months


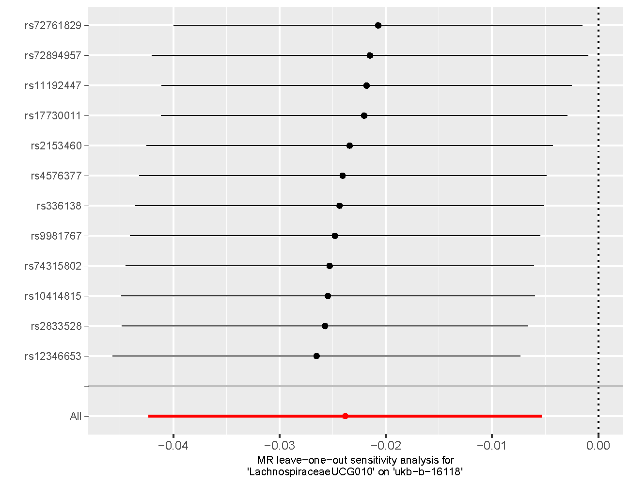

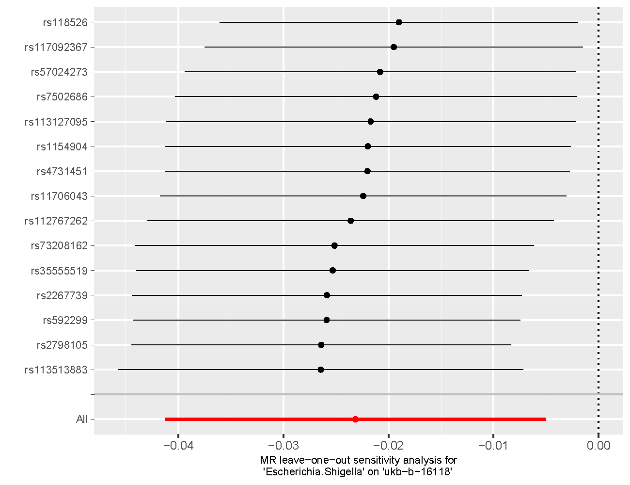


A B


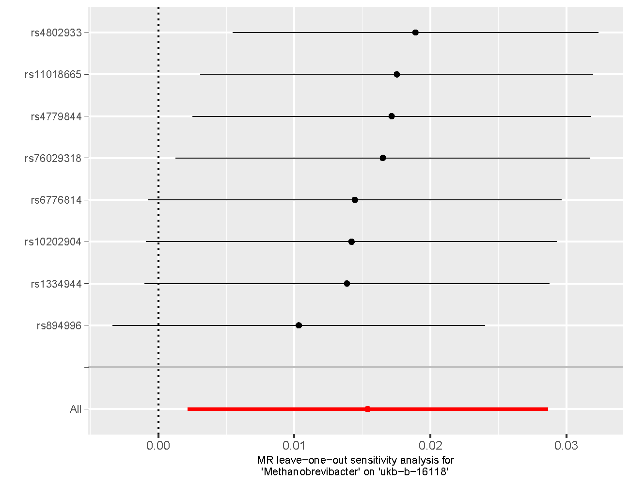

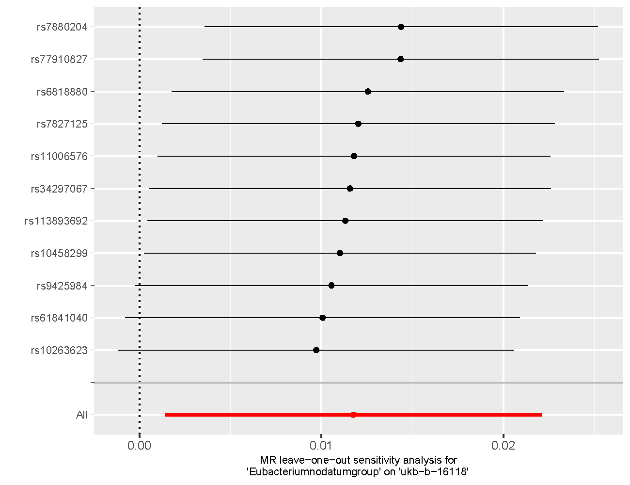


C D


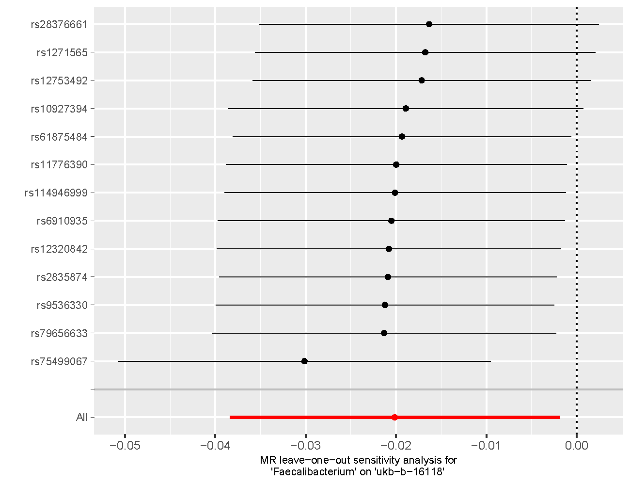

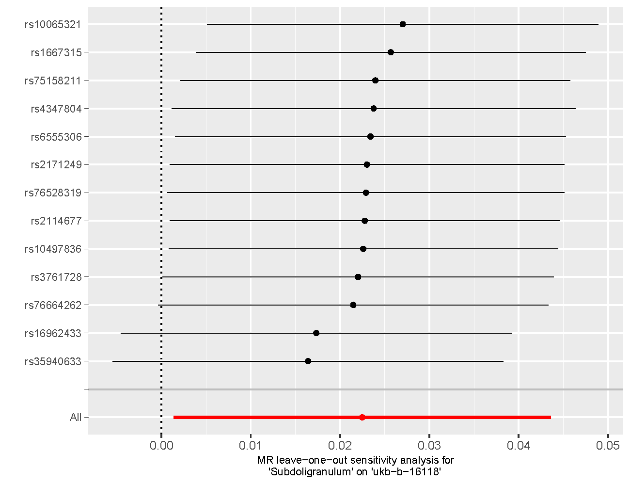


E F

**Supplementary Figure S11**

The stability of Mendelian Randomization (MR) results between exposure (gut microbiota) and the outcome (Thoracic spine pain) was assessed using the leave-one-out method. (A): Butyrivibrio; (B): Ruminococcaceae UCG011; (C): Eubacterium brachy group.


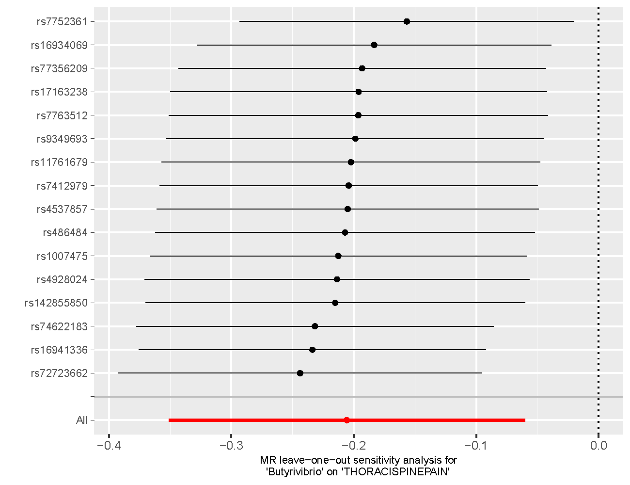

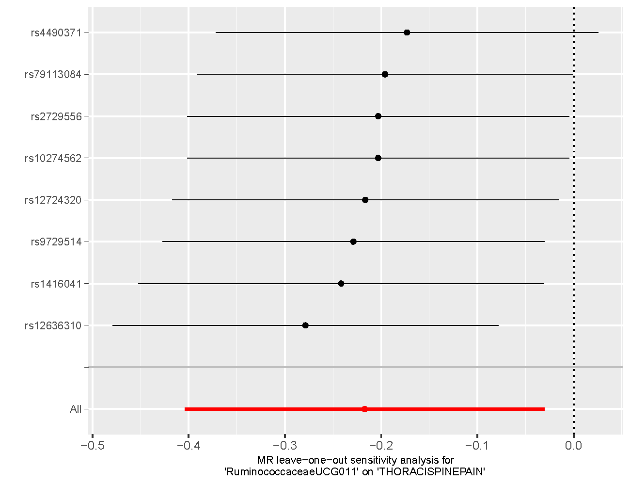


A B


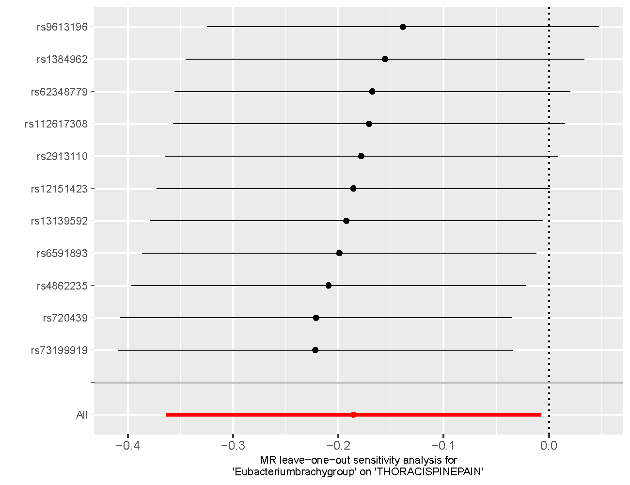


C

**Supplementary Figure S12**

The stability of Mendelian Randomization (MR) results between exposure (gut microbiota) and the outcome (Low back pain) was assessed using the leave-one-out method. (A): Ruminococcaceae UCG011; (B): Oxalobacter; (C): Tyzzerella 3; (D): Olsenella; (E): Eisenbergiella; (F): Roseburia.


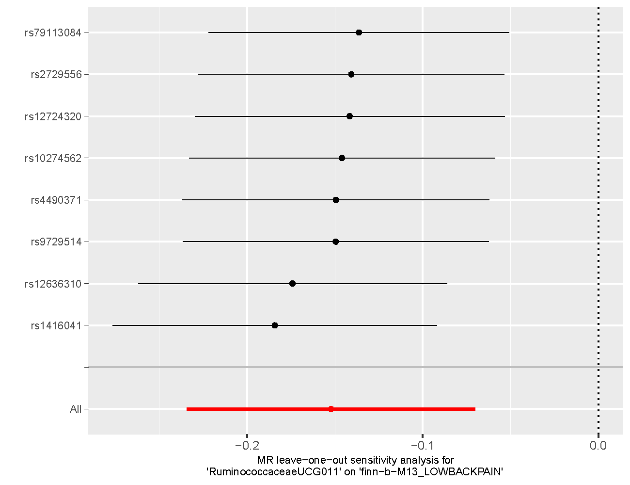

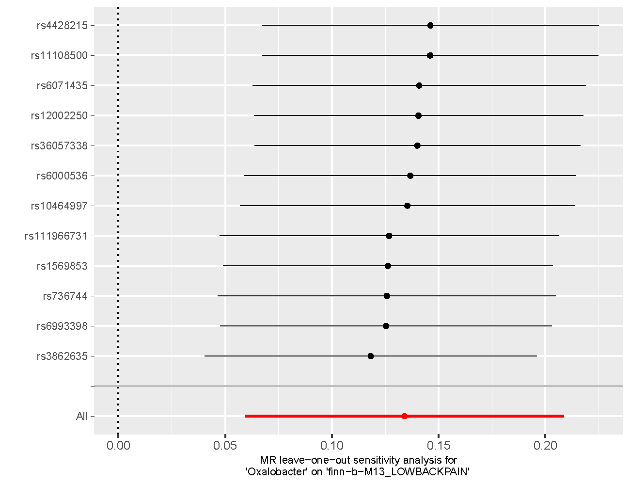


A B


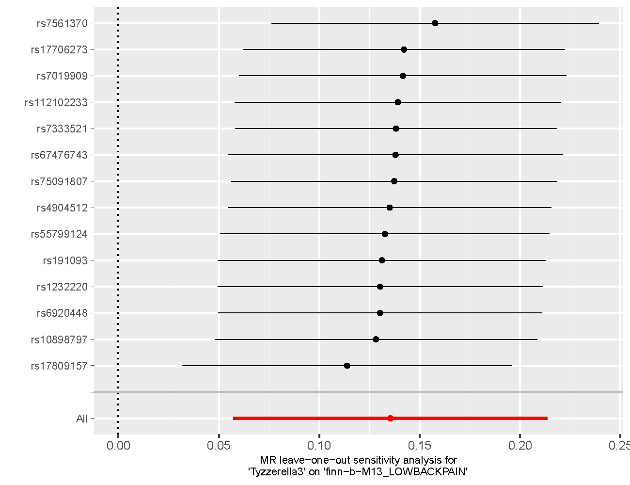

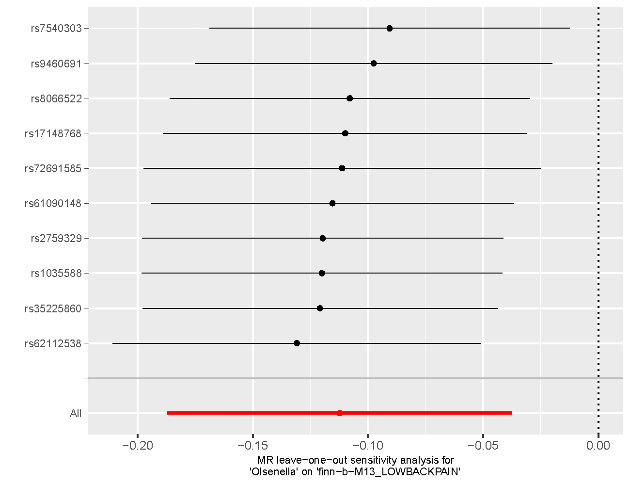


C D


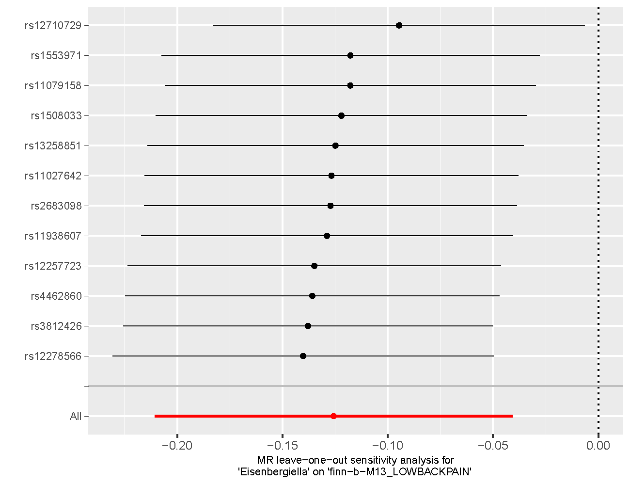

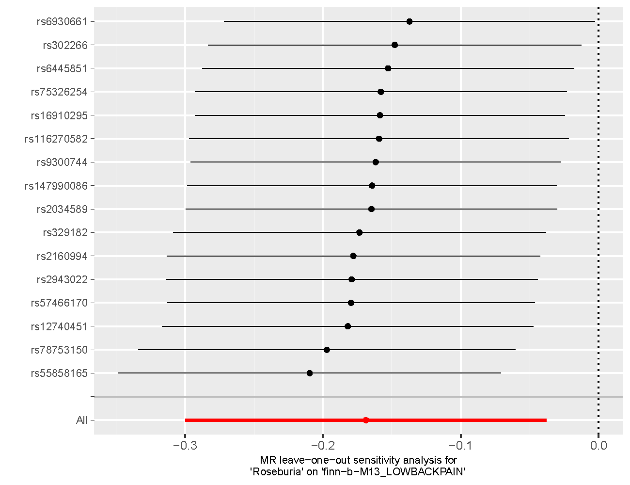


E F

**Supplementary Figure S13**

The stability of Mendelian Randomization (MR) results between exposure (gut microbiota) and the outcome (Lumbar and other intervertebral disk disorders with radiculopathy) was assessed using the leave-one-out method. (A): Ruminococcus gnavus group; (B): Eubacterium hallii group; (C): Rikenellaceae RC9 gut group; (D): Prevotella 7; (E): Lachnoclostridium; (F): Oscillibacter; (G): Eubacterium oxidoreducens group.

ukb-b-18279: Lumbar and other intervertebral disk disorders with radiculopathy


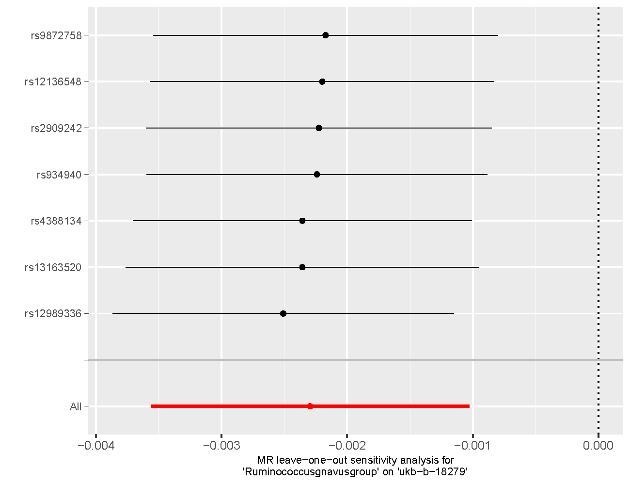

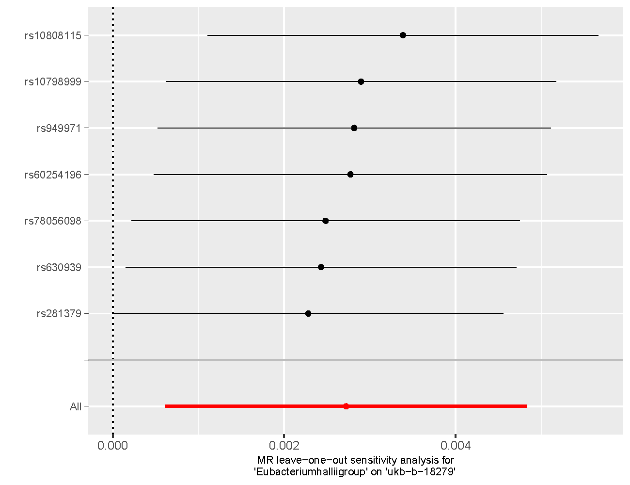


A B


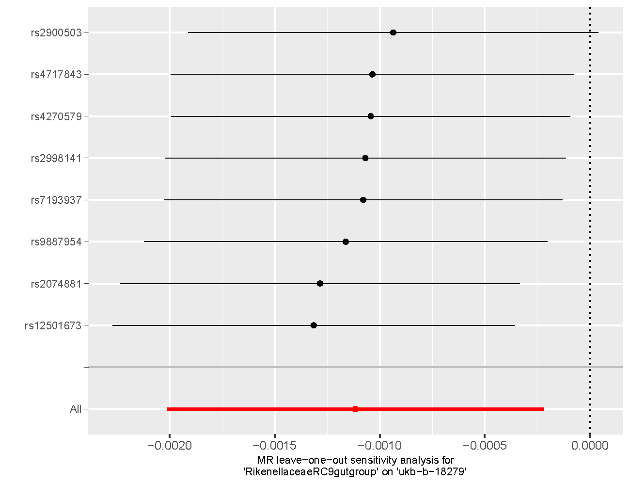

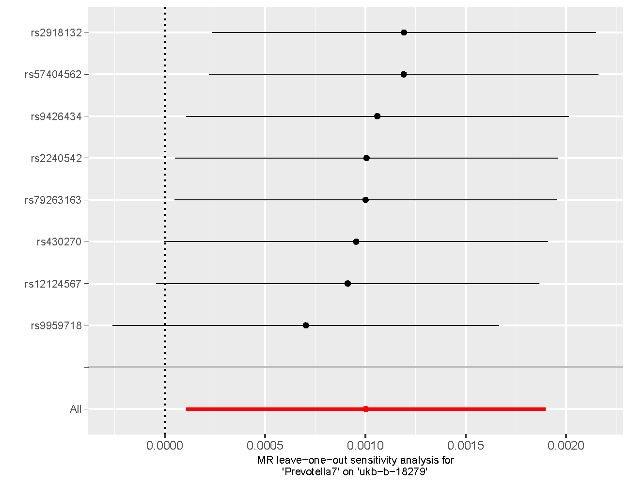


C D


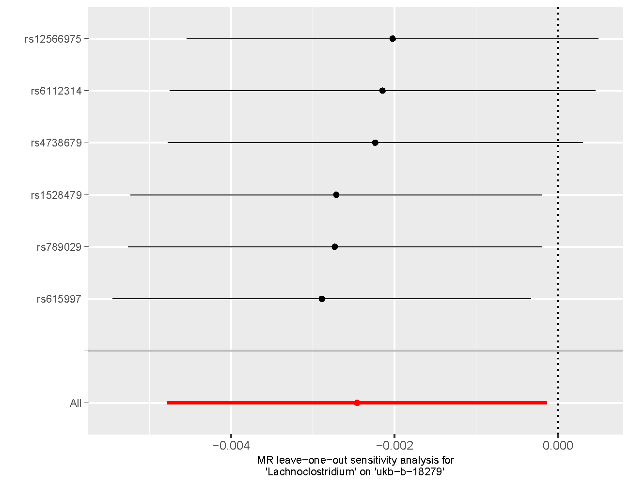

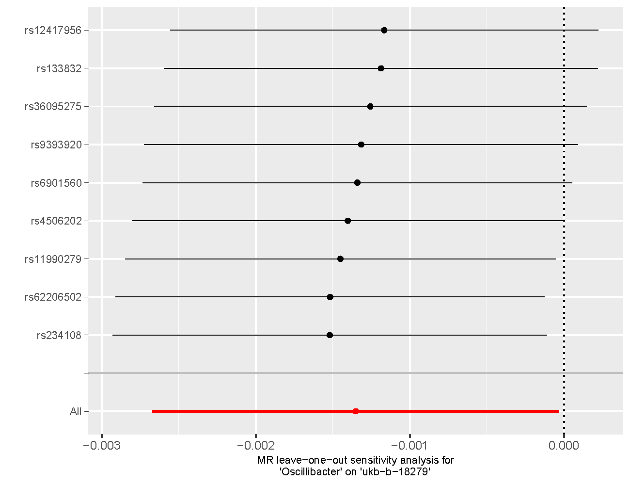


E F


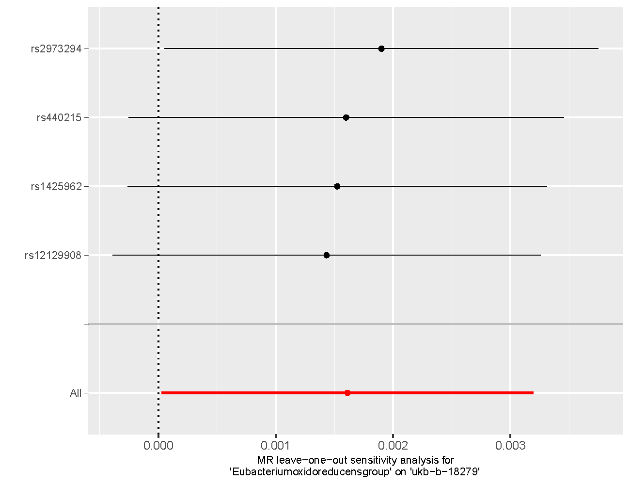


G

**Supplementary Figure S14**

The stability of Mendelian Randomization (MR) results between exposure (gut microbiota) and the outcome (Lower back pain or/and sciatica) was assessed using the leave-one-out method. (A): Olsenella; (B): Ruminococcaceae UCG011; (C): Tyzzerella 3; (D): Eisenbergiella; (E): Oxalobacter; (F): Eubacterium fissicatena group; (G): Turicibacter; (H): Eubacterium nodatum group; (I): Lachnospiraceae NK4A136 group; (J): Ruminiclostridium 5; (K): Oscillibacter.


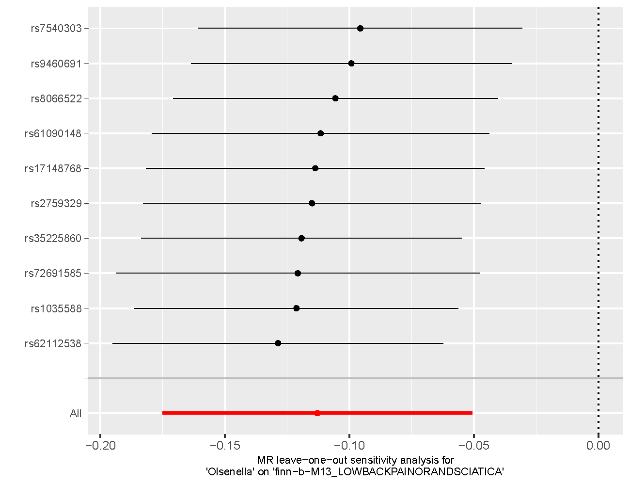

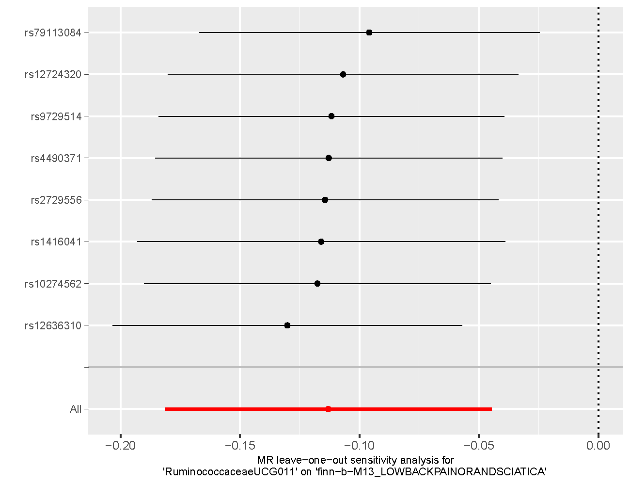


A B


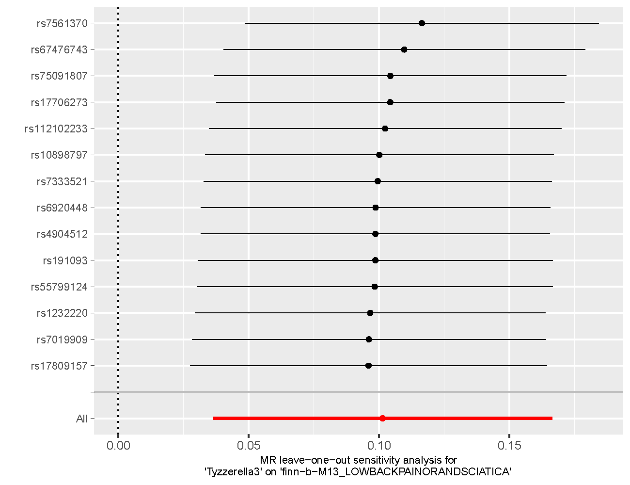

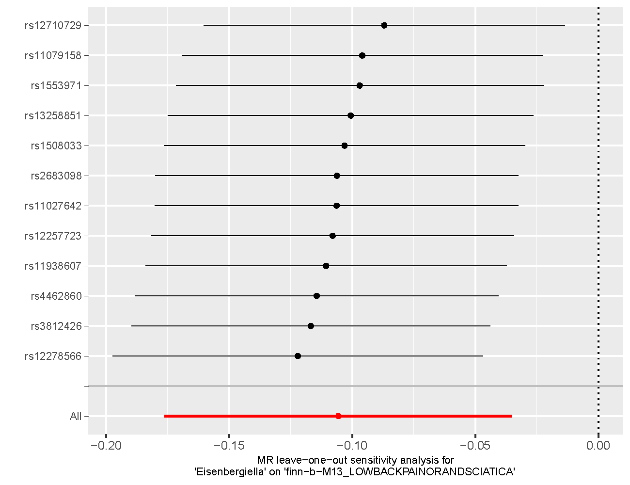


C D


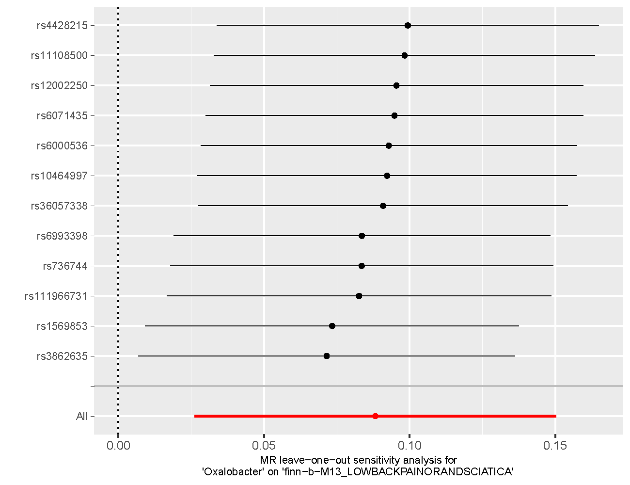

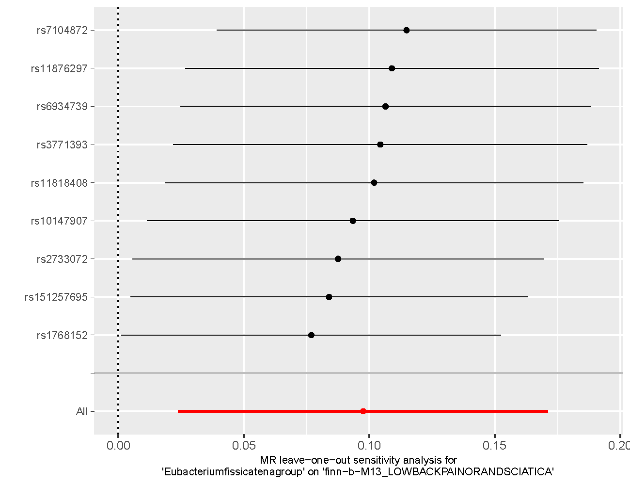


E F


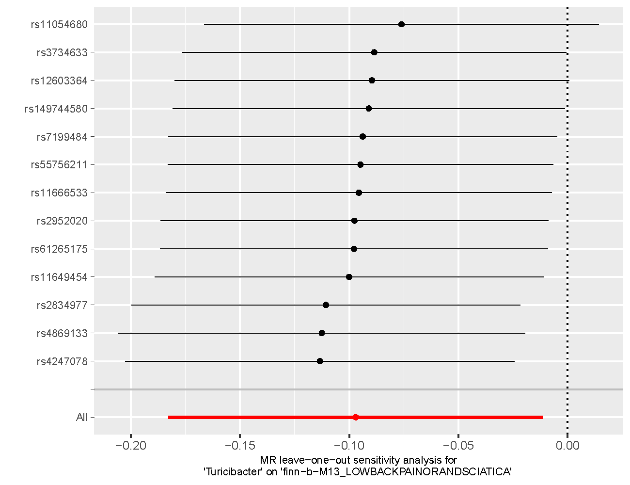

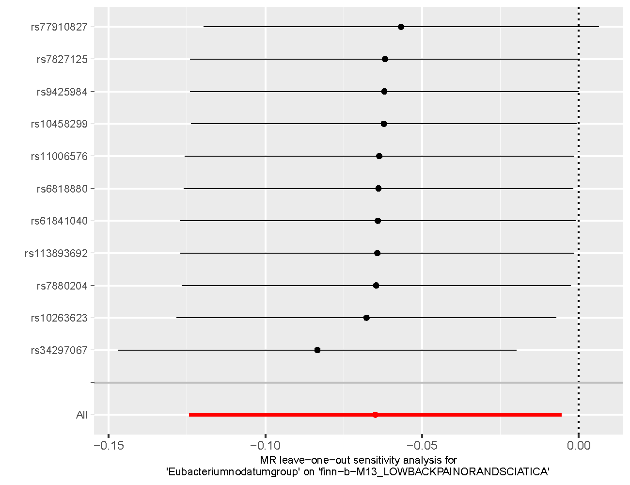


G H


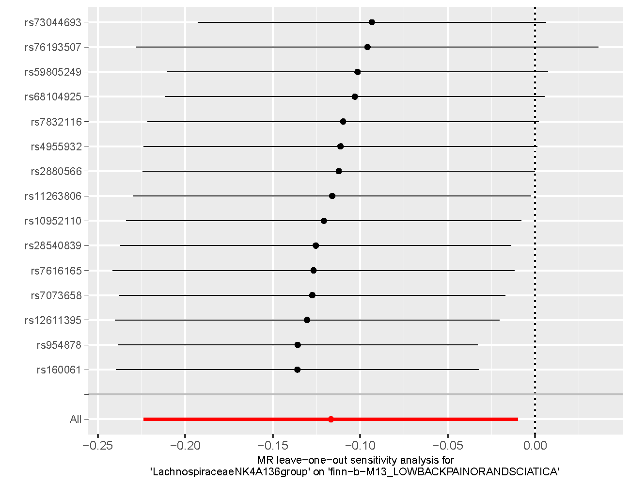

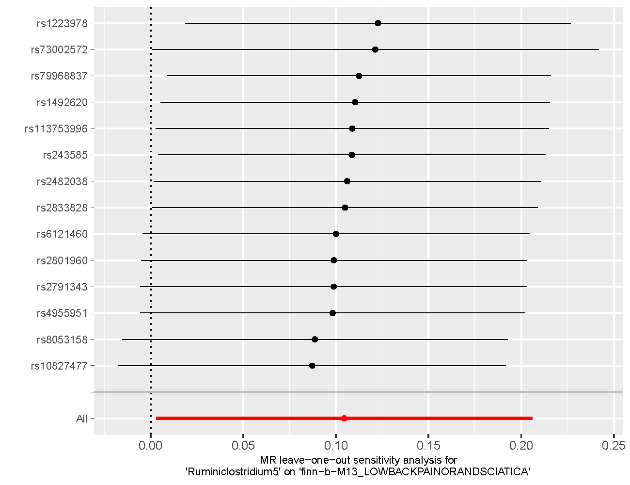


I J


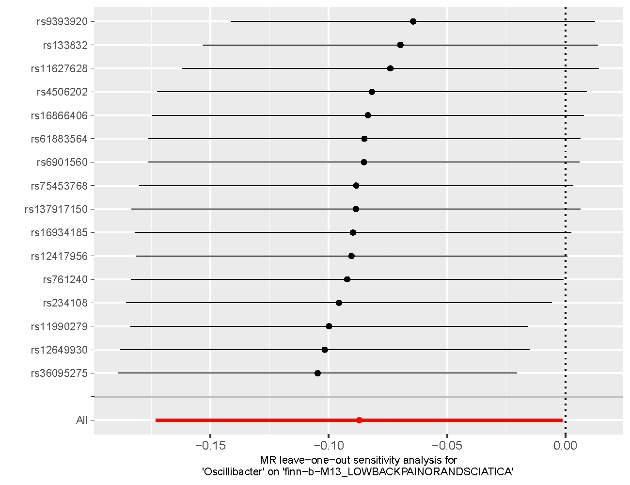


K

**Supplementary Figure S15**

The stability of Mendelian Randomization (MR) results between exposure (gut microbiota) and the outcome (Back pain in the last month) was assessed using the leave-one-out method. (A): Alloprevotella; (B): Christensenellaceae R.7 group; (C): Intestinibacter; (D): Eubacterium hallii group; (E): Lachnoclostridium; (F): Oscillospira; (G): Ruminococcaceae UCG010.

ukb-b-9838: Back pain in the last month。


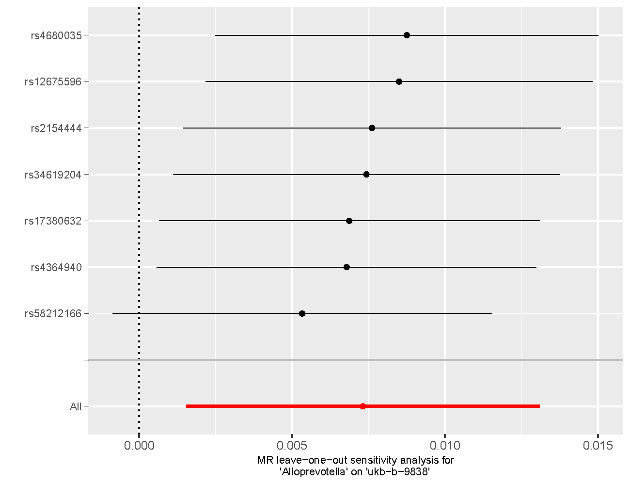

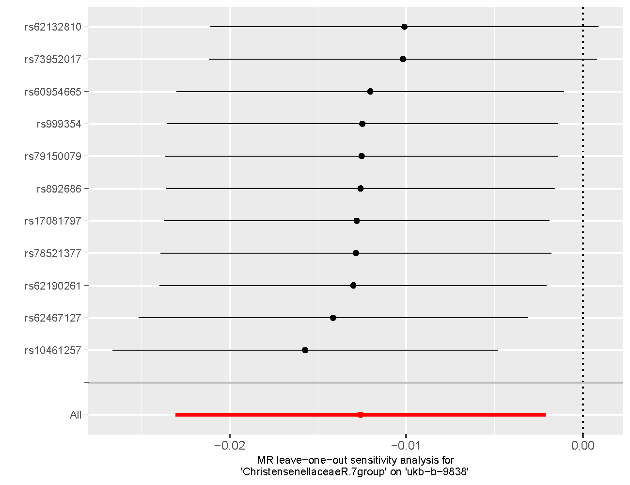


A B


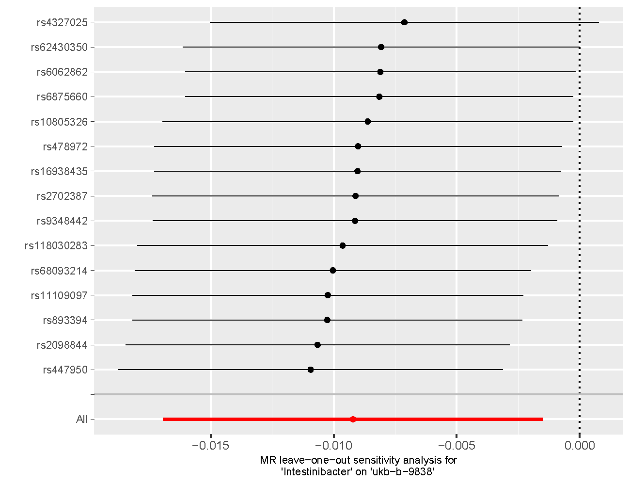

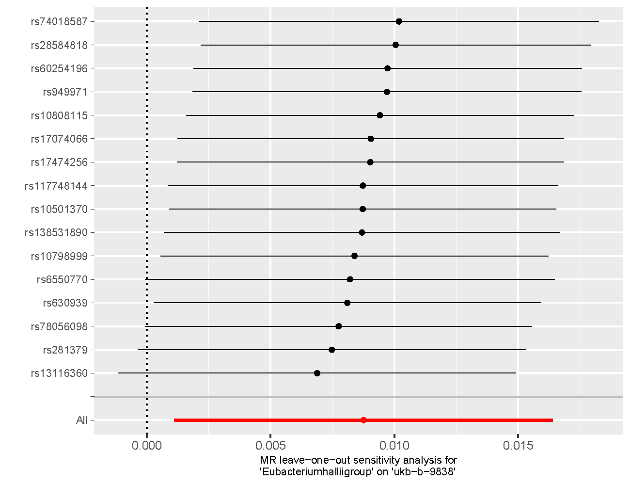


C D


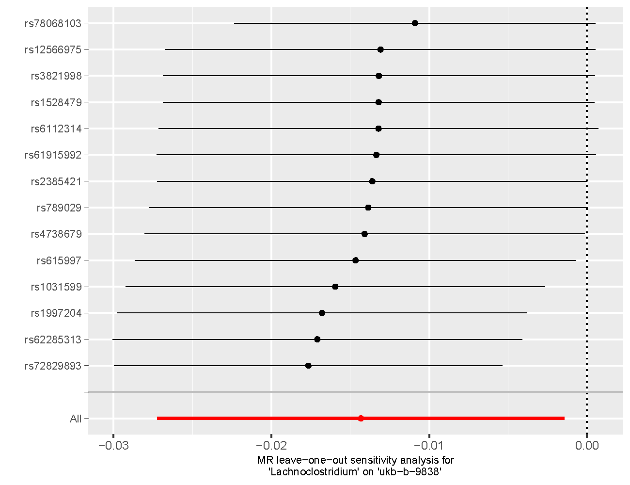

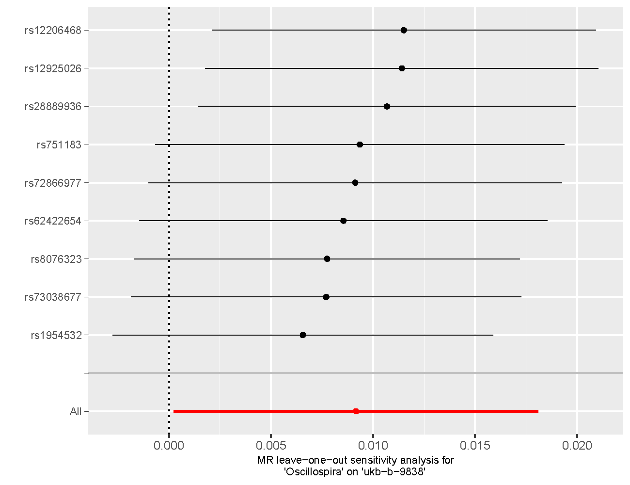


E F


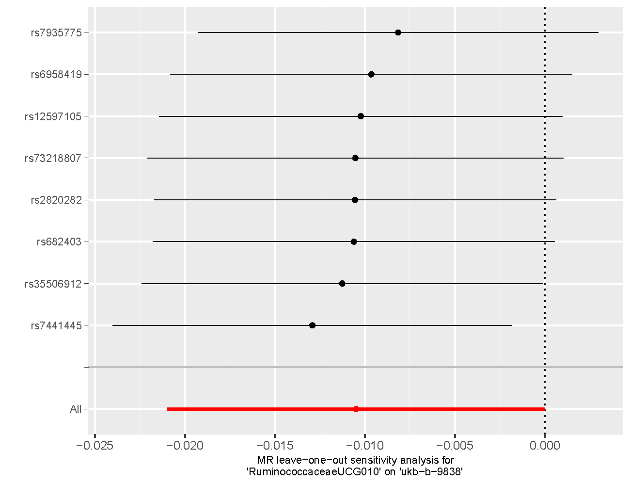


G

**Supplementary Figure S16**

The stability of Mendelian Randomization (MR) results between exposure (gut microbiota) and the outcome (Back pain for 3+ months) was assessed using the leave-one-out method. (A): Coprococcus 2; (B): Ruminococcus 1.

ukb-b-8463: Back pain for 3+ months


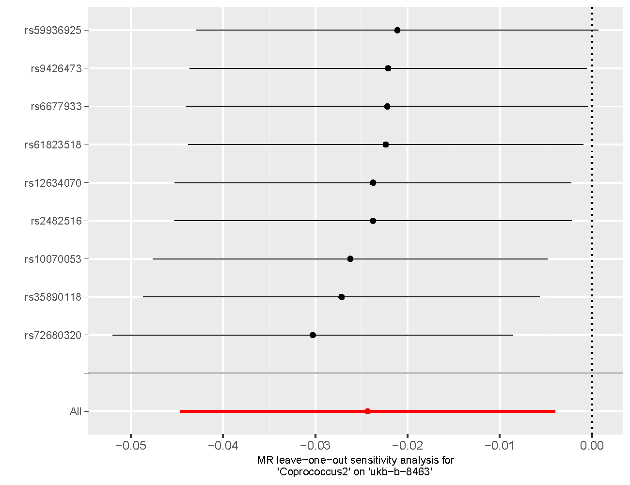

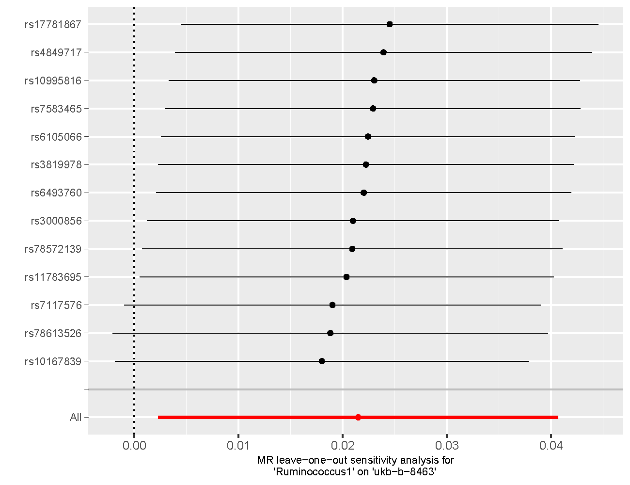


A B
