# Supplementary material for: Genetically predicted causal effects of gut microbiota on spinal pain: a two-sample Mendelian randomization analysis
Source: Front Microbiol. 2024 Mar 25;15:1357303. doi: 10.3389/fmicb.2024.1357303 (PMC10999687; doi:10.3389/fmicb.2024.1357303)
Supplement: Supplementary Data Sheet 3 — Analysis codes for MR analysis. [file Data_Sheet_3.docx]

**Analysis codes for MR analysis**

exposureFile="exposure.F.csv"

outcomeFile="ukb-b-18596.vcf.gz"

outcomeName=" ukb-b-18596"

setwd("C:\\Users\\84290\\Desktop\\179.gutMR\\07.MR")

exposure_dat=read_exposure_data(filename=exposureFile,

sep = ",",

snp_col = "SNP",

beta_col = "beta.exposure",

se_col = "se.exposure",

pval_col = "pval.exposure",

effect_allele_col="effect_allele.exposure",

other_allele_col = "other_allele.exposure",

eaf_col = "eaf.exposure",

phenotype_col = "exposure",

samplesize_col = "samplesize.exposure",

chr_col="chr.exposure", pos_col = "pos.exposure",

clump=FALSE)

vcfRT=readVcf(outcomeFile)

outcomeData=gwasvcf_to_TwoSampleMR(vcf=vcfRT, type="outcome")

outcomeTab=merge(exposure_dat, outcomeData, by.x="SNP", by.y="SNP")

write.csv(outcomeTab[,-(2:ncol(exposure_dat))], file="outcome.csv")

outcome_data=read_outcome_data(snps=exposure_dat$SNP,

filename="outcome.csv", sep = ",",

snp_col = "SNP",

beta_col = "beta.outcome",

se_col = "se.outcome",

effect_allele_col = "effect_allele.outcome",

other_allele_col = "other_allele.outcome",

pval_col = "pval.outcome",

eaf_col = "eaf.outcome")

outcome_data$outcome=outcomeName

dat=harmonise_data(exposure_dat, outcome_data)

outTab=dat[dat$mr_keep=="TRUE",]

write.csv(outTab, file="table.SNP.csv", row.names=F)

mrResult=mr(dat)

mrTab=generate_odds_ratios(mrResult)

write.csv(mrTab, file="table.MRresult.csv", row.names=F)

heterTab=mr_heterogeneity(dat)

write.csv(heterTab, file="table.heterogeneity.csv", row.names=F)

pleioTab=mr_pleiotropy_test(dat)

write.csv(pleioTab, file="table.pleiotropy.csv", row.names=F)

pdf(file="pic.scatter_plot.pdf", width=7.5, height=7)

mr_scatter_plot(mrResult, dat)

dev.off()

res_single=mr_singlesnp(dat)

pdf(file="pic.forest.pdf", width=7, height=5.5)

mr_forest_plot(res_single)

dev.off()

pdf(file="pic.funnel_plot.pdf", width=7, height=6.5)

mr_funnel_plot(singlesnp_results = res_single)

dev.off()

pdf(file="pic.leaveoneout.pdf", width=7, height=5.5)

mr_leaveoneout_plot(leaveoneout_results = mr_leaveoneout(dat))

dev.off()

mrFile="table.MRresult.csv"

pleFile="table.pleiotropy.csv"

setwd("C:\\Users\\84290\\Desktop\\179.gutMR\\08.forest")

rt=read.csv(mrFile, header=T, sep=",", check.names=F)

ivw=rt[((rt$method=="Inverse variance weighted") & (rt$pval<0.05)),]

pleRT=read.csv(pleFile, header=T, sep=",", check.names=F)

pleRT=pleRT[pleRT$pval>0.05,]

gutLists=as.vector(pleRT$exposure)

outTab=ivw[ivw$exposure %in% gutLists,]

write.csv(outTab, file="IVW.filter.csv", row.names=F)
